# γδ T cell antigen receptor polyspecificity enables T cell responses to a broad range of immune challenges

**DOI:** 10.1073/pnas.2315592121

**Published:** 2024-01-16

**Authors:** Jing Guo, Roshni Roy Chowdhury, Vamsee Mallajosyula, Jianming Xie, Megha Dubey, Yuanyuan Liu, Jing Li, Yu-ling Wei, Brad A. Palanski, Conghua Wang, Lingfeng Qiu, Mané Ohanyan, Oliver Kask, Elsa Sola, Lilit Kamalyan, David B. Lewis, Thomas J. Scriba, Mark M. Davis, Dylan Dodd, Xun Zeng, Yueh-hsiu Chien

**Affiliations:** ^a^Department of Microbiology and Immunology, Stanford University School of Medicine, Stanford, CA 94305; ^b^Program in Immunology, Stanford University School of Medicine, Stanford, CA 94305; ^c^Institute for Immunity, Transplantation and Infection, Stanford University School of Medicine, Stanford, CA 94305; ^d^Department of Pathology, Stanford University School of Medicine, Stanford, CA 94305; ^e^Department of Chemistry, Stanford University, Stanford, CA 94305; ^f^State Key Laboratory for Diagnosis and Treatment of Infectious Diseases, National Clinical Research Center for Infectious Diseases, The First Affiliated Hospital, College of Medicine, Zhejiang University, Hangzhou 310003, China; ^g^National Medical Center for Infectious Diseases, Collaborative Innovation Center for Diagnosis and Treatment of Infectious Diseases, The First Affiliated Hospital, College of Medicine, Zhejiang University, Hangzhou 310003, China; ^h^Department of Pediatrics, Stanford University School of Medicine, Stanford, CA 94305; ^i^South African Tuberculosis Vaccine Initiative, Institute of Infectious Disease and Molecular Medicine and Division of Immunology, Department of Pathology, University of Cape Town, Cape Town 7700, South Africa; ^j^HHMI, Stanford University School of Medicine, Stanford, CA 94305; ^k^Research Units of Infectious disease and Microecology, Chinese Academy of Medical Sciences, Beijing 100730, China

**Keywords:** γδ T cells, antigen receptor poly-specificity, γδ T cell antigen recognition and response

## Abstract

γδ T cells are essential for immune defense and modulating physiological processes. While they have the potential to recognize large numbers of antigens through somatic gene rearrangement, the antigens which trigger most γδ T cell response remain unidentified, and the role of antigen recognition in γδ T cell function is contentious. Here, we show that some γδ T cell receptors (TCRs) exhibit polyspecificity, recognizing multiple ligands of diverse molecular nature. These ligands include haptens, metabolites, neurotransmitters, posttranslational modifications, as well as peptides and proteins of microbial and host origin. Polyspecific γδ T cells are enriched among activated cells in naive mice and the responding population in infection. They express diverse TCR sequences, have different functional potentials, and include the innate-like γδ T cells, such as the major IL-17 responders in various pathological/physiological conditions. We demonstrate that encountering their antigenic microbiome metabolite maintains their homeostasis and functional response, indicating that their ability to recognize multiple ligands is essential for their function. Human γδ T cells with similar polyspecificity also respond to various immune challenges. This study demonstrates that polyspecificity is a prevalent feature of γδ T cell antigen recognition, which enables rapid and robust T cell responses to a wide range of challenges, highlighting a unique function of γδ T cells.

## Significance

γδ T cells and αβ T cells are conserved throughout vertebrate evolution, indicating that each of these cell types contributes uniquely to maintain host immune competence despite having similar effector functions. Here, we show that some γδ T cells are polyspecific, where the same T cell receptor can recognize multiple ligands of different molecular nature, thus allowing important T cell functions to be induced robustly by a much broader range of ligands and in various physiological and pathological conditions. This contrasts with αβ T cells, which are highly constrained by their requirement to recognize small ligands bound to MHC (major histocompatibility complex) molecules. We propose that γδ T cell polyspecificity is a unique and critical feature of γδ T cell functionality.

γδ T cells and αβ T cells are conserved through vertebrate evolution, indicating that each of these cell types contributes uniquely and is necessary to maintain host immune competence. While classic T cell functions are mostly performed by αβ T cells, γδ T cells are found to have distinct and essential roles in defense against infection and injury (1–3), in autoimmunity (4, 5), cancer (6, 7), and at the neuroimmune interface (8). In addition, γδ T cells serve a critical function in the regulation of physiological functions in the brain (9, 10), the adipose tissue (11, 12), and the intestine (13). Recent work also underscores their potential roles in cancer immune therapy (14, 15) and clinical application against infection (16, 17).

As γδ T cells and αβ T cells produce similar sets of cytokines and both can mount cytolytic responses, the major difference between the two lies in what they are able to recognize and how they are activated. Indeed, γδ T cells and αβ T cells have different antigen recognition requirements and antigen-specific repertoires (1, 18). While αβ TCRs (T cell receptors) recognize peptides or other small molecules bound to major histocompatibility complex (MHC) or MHC-like molecules, γδ TCRs recognize ligands directly without antigen processing and presentation requirements, and the MHCs are not an obligatory component of γδ T cell antigens (1). Moreover, while both αβ and γδ T cells require thymic maturation before entering the periphery, this process greatly limits what αβ T cells can recognize (19, 20), but does not constrain the γδ T cell antigen-specific repertoire (21). Analysis of γδ TCR complementarity determining region 3 (CDR3) sequence diversity and length distributions suggest that γδ T cells have extensive antigen recognition capability, and as a group, γδ TCRs are more similar to immunoglobulins than to αβ TCRs in antigen recognition (1, 22). Despite these advances, the antigens which trigger γδ T cells to initiate a response remain unidentified in most cases.

Moreover, although the majority of the γδ T cells in peripheral lymphoid organs derived from the adult thymus, express diverse TCRs, have a naive phenotype in naive animals, and undergo antigen-driven activation and differentiation, to become effectors (23), the major γδ T cell responders in many different pathological/physiological conditions, are noted for expressing TCRs associated with the innate-like γδ T cells, which have an activated phenotype (CD44^hi^CD62L^−^CD27^−^) in naive animals and the ability to make cytokines rapidly in response to innate immune signals, such as inflammatory cytokines or through pattern recognition receptors without deliberate TCR triggering (24, 25). These cells are of fetal or neonatal origin (26, 27) and express highly focused TCR sequences. It has been proposed that like innate immune cells, TCR engagement is not required for their function (24, 25, 28).

Previously, we found that common B cell antigens, such as phycoerythrin and small organic molecules (haptens) (23, 29), are γδ T cell antigens, recognized directly by specific TCRs. Haptens are small organic chemicals, which, when coupled to proteins, induce an αβ T cell–dependent, hapten-specific antibody response. Although serological responses to haptens were first utilized to illustrate the immune system’s ability to recognize diverse foreign antigens (30), pathogen-derived compounds and chemical modifications of host molecules have structural features similar to haptens. Thus, hapten recognition could serve in pathogen surveillance and the monitoring of altered physiological states. One such example is Cyanine 3 (Cy3), a synthetic molecule with two modified indole groups joined by a polymethine bond. Indole can be produced by microbes (31–33) and is widely distributed in the environment. In addition, tryptophan metabolism generates molecules with indole sidechains. These metabolites play important roles in regulating a variety of physiological responses (34).

In this study, we investigate whether Cy3-specific γδ T cells can recognize naturally produced molecules with structural features similar to Cy3 and find that some Cy3-specific γδ TCRs can recognize indole- and π electron-containing biomolecules with different molecular properties, demonstrating γδ TCR polyspecificity. γδ T cells with this polyspecificity include the innate-like γδ T cells. We demonstrate that signaling through the TCR is required for their response, and encountering their antigenic microbiome metabolite maintains their homeostasis and functional response. Human γδ T cells with the same polyspecificity are also analyzed. Our results underscore the importance of antigen recognition in γδ T cell function and identify γδ T cell polyspecificity as an important feature which enables rapid and robust T cell response to a broad range of pathological and physiological conditions.

## Results

### A Hapten-Specific γδ TCR Recognizes Multiple Ligands of Diverse Molecular Nature.

**Fig. 1.**
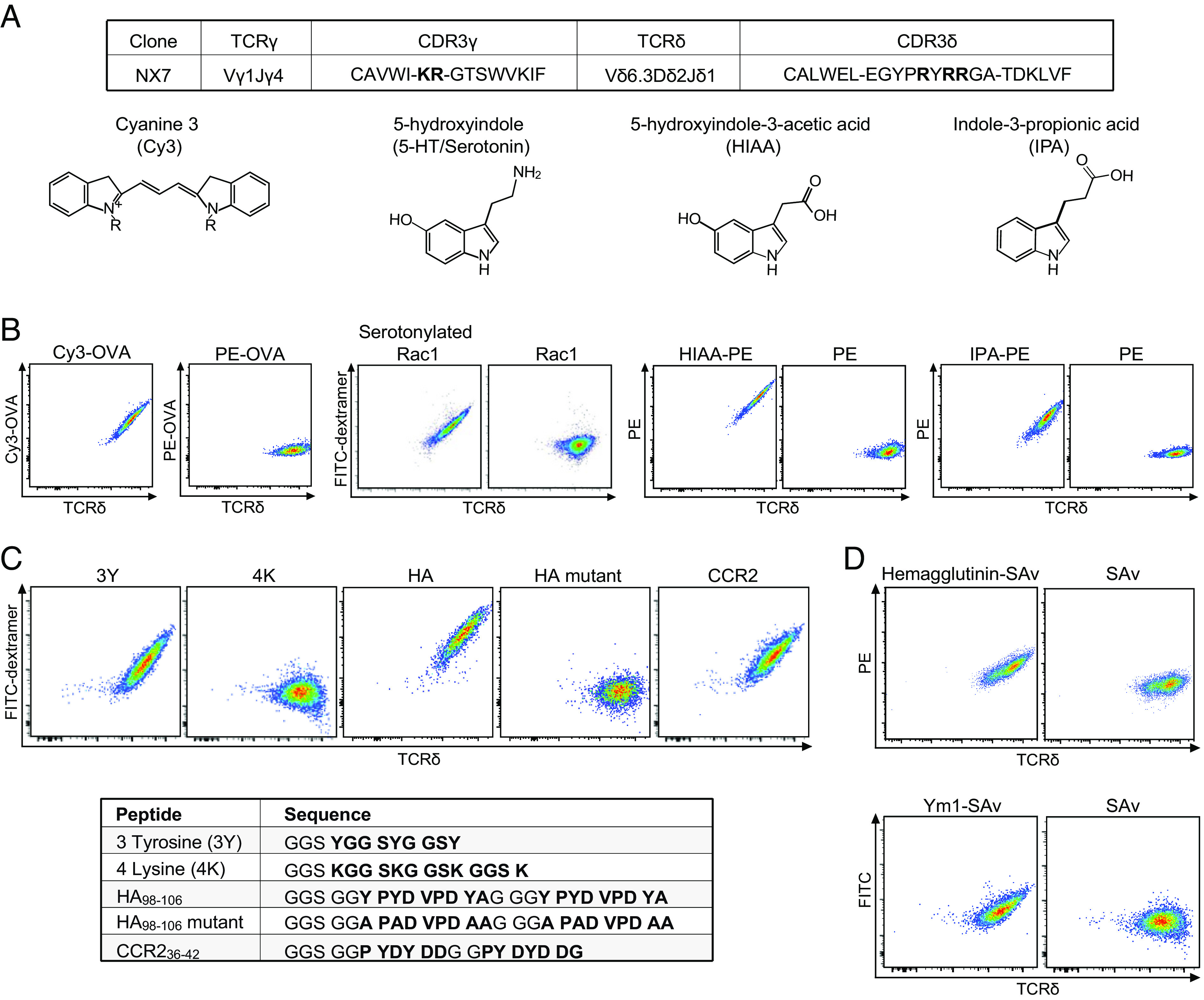
A Cy3-specific γδ TCR recognizes multiple ligands of different molecular nature. (*A*) The NX7 γδ TCR CDR3 sequences (with positively charged amino acid residue in bold) and the chemical structures of Cy3, 5-HT (serotonin), HIAA, and IPA. (*B*–*D*) Representative FACS plots of NX7/58α^−^β^−^ cells stained with (*B*) Cy3-OVA (1 μg/mL), PE-OVA (3 μg/mL), serotonylated or unmodified Rac1 peptide (Biotin-Ahx-GGS GGD TAG QED GGD TAG QED GGD TAG QED, with the Rac1 sequence in bold. Ahx: 6-aminohexanoic acid.) coupled with FITC SAv-dextramer (0.45 μM, SAv concentration), HIAA-PE (1 μg/mL) or PE (1 μg/mL), IPA-PE (10 μg/mL) or PE (10 μg/mL); (*C*) biotinylated 3-tyrosine peptide (3Y), 4-lysine peptide (4K), HA_98-106_ peptide (abbreviated as HA), HA_98-106_ mutant peptide (abbreviated as HA mutant), and murine CCR2_36-42_ peptide (abbreviated as CCR2) coupled with FITC SAv-dextramer (0.45 μM, SAv concentration). The peptide sequences were shown in bold; (*D*) surface cleaved hemagglutinin protein-coupled PE SAv tetramer (2 μM, SAv concentration) or PE-labeled SAv (2 μM, SAv concentration), Ym1 protein-coupled FITC SAv tetramer (10 μM, SAv concentration) or FITC labeled SAv (10 μM, SAv concentration). Hemagglutinin and Ym1 were randomly biotinylated. The results in *B*–*D* were representative of at least three independent experiments.

To test whether Cy3-specific γδ TCRs could recognize indole-containing natural products, we tested a panel of metabolites, commonly produced in the gut, for their ability to compete with Cy3 for binding to a Cy3-specific γδ TCR, NX7 expressed on 58α^−^β^−^ cells. This assay identified 5-hydroxytryptamine (5-HT), commonly known as serotonin, its metabolite, 5-hydroxyindole-3 acetic acid (HIAA), and indole-3-propionic acid (IPA), a metabolite produced exclusively by the microbiota (34), as potential ligands for NX7 TCR (Fig. 1 *A* and *B* and *SI Appendix*, Fig. S1*A*).

Serotonin (5-HT), a neurotransmitter, is primarily found in the gastrointestinal tract, central nervous system, and blood platelets. Serotonylation of small GTPases by transglutaminases is a key step in platelet activation, which leads to the subsequent serotonylation of other granule proteins and their display on platelet surfaces (35). We transamidated the small GTPase peptide Rac1 with serotonin in vitro using enzymatically active recombinant transglutaminase 2 (TG2) (*SI Appendix*, Fig. S1*B*) and found that the serotonylated, but not the unmodified, Rac1 peptide was recognized by NX7/58α^−^β^−^ cells (Fig. 1*B*). Additionally, HIAA and IPA, when coupled to carrier proteins, stained, and activated NX7/58α^−^β^−^ cells (Fig. 1*B* and *SI Appendix*, Fig. S1 *C* and *D*).

Indole is an aromatic heterocyclic compound and the CDR3 of NX7 TCR gamma and delta chain consists of multiple positively charged amino acid residues (Fig. 1*A*), suggesting potential involvement of cation-π electron interactions (36) in this TCR-ligand binding. Consistent with this supposition, the neurotransmitters dopamine and epinephrine competed with Cy3 for NX7 TCR binding (*SI Appendix*, Fig. S1*A*) and the NX7 TCR recognized the synthetic 3-Tyr peptide (GSSYGGSYGGSY), the hemagglutinin peptide HA_98-106_ (YPYDVPDY) (abbreviated as HA here), a sequence within the antigenic target for a group of broadly protective antibodies (37), and the N-terminal extracellular C-C motif chemokine receptor 2 peptide CCR2_36-42_ (PYDYDDG) (Fig. 1*C*). In contrast, NX7 did not recognize the 4-Lys peptide (GSSKGGSKGGSKGGSK), nor the mutant HA_98-106_ (abbreviated as HA mutant here) where all the Tyr residues were substituted with Alanine (A) and thus lacked π electrons (Fig. 1*C*). Additionally, NX7 recognized bromelain-cleaved, purified hemagglutinin protein from influenza H1N1 PR8/1934 (PR8) virus, and a chitinase-like protein Ym1 which has a solvent-exposed pocket with Trp, Tyr, and Asp residues (38) (Fig. 1*D*). Ym1 induction has been associated with various inflammatory conditions and γδ T cell expansion (39).

Although the structural basis for these TCR-ligand interactions will require further analyses, these observations indicated γδ TCR polyspecificity, in that a specific γδ TCR can recognize a broad array of ligands from small chemicals to posttranslational modification, along with peptides and proteins generated in different physiological/pathological settings and of host and microbial origin.

### Polyspecific γδ T Cells Are Enriched among Cells with an Activated Phenotype in Naïve Mice and Have Various Effector Function Potentials.

**Fig. 2.**
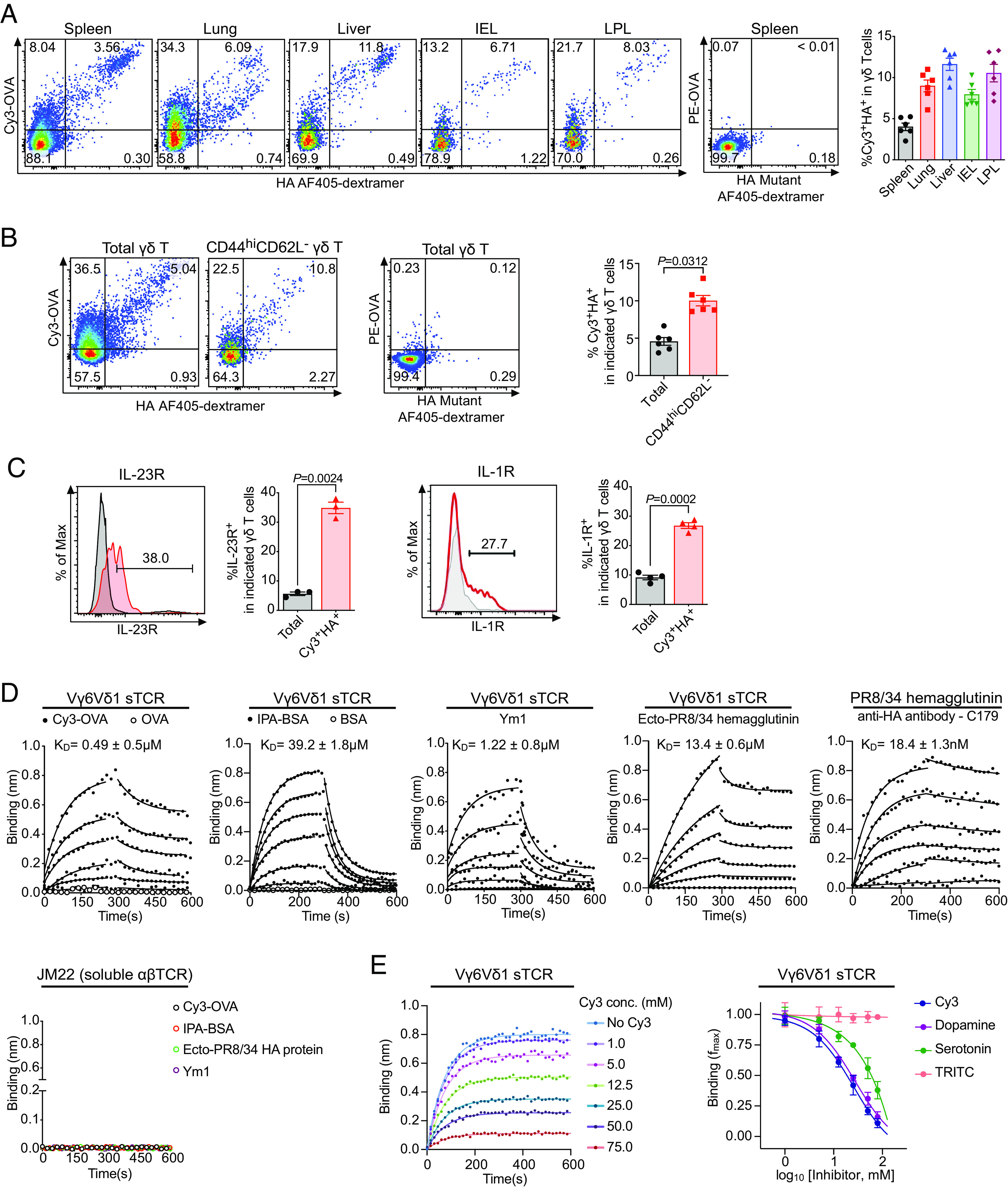
Polyspecific γδ T cells are enriched in activated cells in naive mice and include the innate-like Vγ6Vδ1 cells. (*A*–*C*) A representative FACS plot of (*A*) Cy3 and HA staining of total γδ T cells from spleen, lung, liver, intestinal epithelium (IEL), and lamina propria (LPL) of naive C57BL/6 mice (*Left*), PE-OVA and HA mutant staining of splenic γδ T cells (*Middle*), and the frequencies of Cy3^+^HA^+^ cells among total γδ T cells in each organ (*Right*). (*B*) Cy3 and HA staining of total γδ T and CD44^hi^CD62L^-^ γδ T cells from spleens of naive C57BL/6 mice (*Left*), PE-OVA and HA mutant staining of splenic γδ T cells (*Middle*), and the frequencies of Cy3^+^HA^+^ cells among indicated γδ T cells (*Right*); (*C*) IL-23R staining on total γδ T cells (gray line) and Cy3^+^HA^+^ γδ T cells (red line) from the spleen of naive IL-23R reporter mice (*Top*) and IL-1R staining on total γδ T cells (gray line) and Cy3^+^HA^+^ γδ T cells (red line) from the spleen of naive C57BL/6 mice (*Bottom*), and the percentage of IL-23R^+^ and IL-1R^+^ cells in total γδ T cells and Cy3^+^HA^+^ γδ T cells. Enriched γδ T cells from each organ were stained with Cy3-OVA (2 μg/mL), PE-OVA (2 μg/mL), HA coupled AF405-dextramer (1.35 μM), and HA mutant coupled AF405-dextramer (1.35 μM). Each data point represented the result from an individual mouse. Summary results are graphed as the mean ± SEM. The *P* values in *C* were determined using a two-tailed, paired *t* test. (*D*) Binding kinetics and dissociation constants (K_D_) of soluble Vγ6Vδ1 TCR with Cy3-OVA, OVA, IPA-BSA (indole-3-propionic acid conjugated bovine serum albumin), BSA, Ym1, and the fusion-competent ectodomain of influenza virus A/PR8/34 (Ecto-PR8/34) hemagglutinin as determined by biolayer interferometry (BLI). The conformation of the fusion-competent ectodomain of PR/34 HA was confirmed by the binding of HA conformation-specific antibody, C179, as control. Graphs showed overlays of binding traces at 20 μM followed by twofold dilutions of the analytes with the Vγ6Vδ1 TCR, and binding traces at 100 nM followed by twofold dilutions of the analyte with C179 binding. The data points were represented as circles, and the fits were indicated by solid lines. An irrelevant αβ TCR JM22 was served as control. The data shown were representative traces from one of three independent experiments. (*E*) Representative traces of Ym1 binding to soluble Vγ6Vδ1 TCR in the presence of increasing concentration of Cy3 (*Left*) and the competition of Ym1 binding to soluble Vγ6Vδ1 TCR in the presence of increasing concentration of Cy3 (*Left*) and by indicated small molecules (*Right*) as determined by biolayer interferometry. The fractional reduction in maximal Ym1 binding (*f*_max_) as a function of increasing concentration of the indicated small molecules was plotted. As shown, the negative control TRITC (Tetramethylrhodamine) did not inhibit Ym1 binding. Mean ± SD was shown for three independent experiments.

To evaluate the extent of γδ T cell polyspecificity, we stained splenic γδ T cells from naive mice with Cy3-OVA, HIAA-APC (allophycocyanin), IPA-APC, and AF405 labeled HA dextramer. We found that ~10 to 15% of total splenic γδ T cells were Cy3^+^, of which ~70% costained with HIAA or IPA, but only~30% costained with HA (Fig. 2*A* and *SI Appendix*, Fig. S2 *A–D*). These results indicated the heterogeneity of Cy3-specific γδ TCRs in different ligand binding.

We then used Cy3 and HA costaining as a criterion to examine the abundance of polyspecific γδ T cells in different organs of naive mice. We found that ~4% of the splenic γδ T cells and ~7 to 12% of the γδ T cells in the lung, liver, intestinal intraepithelium (IEL), and lamina propria (LPL) were polyspecific (Fig. 2*A*).

In naïve mice spleens, Cy3^+^HA^+^ γδ T cells constituted ~5% of total γδ T cells, but ~10% of CD44^hi^CD62L^−^(activated) γδ T cells (Fig. 2*B*). ~20% of Cy3^+^HA^+^ γδ T cells have an activated phenotype: CD44^hi^CD62L^-^CD27^-^, as compared to ~9% of total γδ T cells (*SI Appendix*, Fig. S2 *E* and *F*). Furthermore, ~30% of Cy3^+^HA^+^ γδ T cells as compared to ~5% of total γδ T cells expressed receptors for the inflammatory cytokines IL-1 and IL-23 (Fig. 2*C*). The expression of the IL-1 and IL-23 receptors on γδ T cells has been attributed to prior antigenic encounter and the subsequent ability to respond to environmental cues (23). These observations suggested that a substantial fraction of polyspecific γδ T cells in the periphery have encountered ligands in naive animals.

To better understand the functional potential of polyspecific γδ T cells, we carried out single-cell RNA-seq on splenic Cy3^+^HA^+^ γδ T cells isolated from naive C57BL/6 mice using Smart-seq2 protocol (40). Unsupervised clustering of these Cy3^+^HA^+^ γδ T cells identified three clusters. Cluster 1 displayed naive signatures with high expression of Sell (CD62L), Lef1, and CD27. Cells in cluster 2 had the potential to become IFNγ-producing cells (expressing Tbx21). Cluster 3 showed higher expression of genes associated with IL-17 production (Rorc, Il23r, and Maf) (*SI Appendix*, Fig. S3). This result indicated the functional heterogeneity of polyspecific γδ T cells.

### Polyspecific γδ TCR Sequences Are Heterogeneous and Include Those Expressed by the Innate-Like γδ T Cells.

To identify the characteristics of Cy3^+^HA^+^ γδ TCRs, we analyzed splenic γδ T cells from naive mice using barcode-enabled directly ex vivo single-cell TCR sequencing (scTCR-seq) (41, 42). For comparison, we also determined the TCR sequences from Cy3^−^HA^−^ γδ T cells and total spleen γδ T cells (Datasets S1–S3). We found that the majority of Cy3^+^HA^+^ γδ TCRs utilized different Vγ and Vδ gene segments and had diverse CDR3 sequences and length distributions (Dataset S1). Some of these CDR3 sequences, like the NX7 TCR, were enriched in positively charged residues, while others were enriched in residues that confer flexibility, such as Gly and Ser.

Several TCRs expressed on innate-like γδ T cells are among the Cy3^+^HA^+^, but not the Cy3^−^HA^−^ γδ TCRs. These include Vγ1Jγ1 (--CAVW(I)-(X)-(G)TSWVKIF--) paired with Vα15N-1(Vδ6.3) Dδ2Jδ1 (--CAWE(L)(X)-(I)GGIR(A)-(T)DKLVF--) [nomenclature according to Heilig and Tonegawa (43)] (Dataset S1), noted for expressing on innate-like γδ T cells, which rapidly produce IFNγ and/or IL-4 (26). Similar TCR sequences have been described for Thy1^lo^ γδ thymocytes and their peripheral counterparts (44) and for γδ TCR clones that respond to mycobacterial-purified protein derivative (PPD) (45), heat shock protein-derived peptide (46), synthetic copolymers of glutamic acid and tyrosine (47), as well as cardiolipin and related anionic phospholipids (48). We expressed the γδ TCRs of a PPD-specific clone BNT-9.12 (45) in 58α^−^β^−^ cells and demonstrated their specificity to Cy3 and HA (*SI Appendix*, Fig. S4).

In addition, multiple Cy3^+^HA^+^ γδ T cells expressed TCRs encoded by Vγ6Jγ1 chain (--CACWD-SSGFH--) paired with Vδ1Dδ2Jδ2 chain (--CGSD-IGG-SSWDTR--), with no N-nucleotide additions at the CDR3 junctions (abbreviated as Vγ6Vδ1 TCR). γδ T cells express this canonical TCRs are generated during fetal development (27). They are the major cell population poised to make IL-17 rapidly in naive mice (42) and respond to different pathological challenges and physiological conditions at different anatomical sites (3, 6, 9–12, 49). Although the Vγ6Vδ1 γδ T cells are commonly designated as innate-like γδ T cells, surface TCR expression in the periphery is necessary for their function in cases that are examined (6, 10).

To confirm the polyspecificity of Vγ6Vδ1 TCR, we generated a soluble form of this TCR and demonstrated its direct binding to Cy3-OVA and IPA-BSA but not to OVA or BSA. Additionally, this TCR directly bound a recombinant ectodomain of hemagglutinin from influenza virus PR8 and Ym1 (Fig. 2*D*). The recombinant HA protein was in its native conformation, as it bound an HA conformation specific antibody (C179) with K_D_ = 18.4 nM. We also demonstrated that Cy3, dopamine, and serotonin, but not tetramethyl-rhodamine could compete with Ym1 for Vγ6Vδ1 TCR binding (Fig. 2*E*).

Taken together, polyspecific γδ TCRs appeared to share features described for polyreactive antibodies (50), which include natural antibodies, secreted by fetal-derived B1 cells in the absence of deliberate foreign antigen exposure. Their antibody sequence repertories are of limited diversity, with few V gene combinations and much reduced, or nonexistent, N-region addition. These antibodies provide an essential first line of defense. Some somatically hypermutated, isotype-switched antibodies are also polyspecific, such as the anti-HIV neutralizing antibodies. While certain polyreactive antibodies are enriched in positively charged amino acid residues or have long, flexible, hydrophobic IgH CDR3s, these features are not predictive of polyspecificity.

### Commensal Microbiota Metabolite IPA Maintains Polyspecific Vγ6Vδ1 T Cell Homeostasis.

Numerous studies indicated that commensal microflora modulating γδ T cell development and function. Several studies also indicated that the microbiota influences the accumulation and the IL-17 response of the Vγ6Vδ1 γδT cells (3, 6, 10, 51). In addition, it was reported the microbial-driven expansion of CD62L^−^IL-1R1^+^ Tγδ17 cells requires the expression of VAV1 but not Toll-like receptors or antigen presentation pathways in γδ T cells (52). VAV-1, a guanine nucleotide exchange factor, is required for γδ T cell activation through the TCR (53).

As we showed that the polyspecific γδ TCRs recognized the microbiome metabolite IPA, we asked whether IPA regulated the homeostasis of these cells in vivo focusing on the analysis of Vγ6Vδ1 γδT cells, as they are the major responders in various pathological/physiological conditions (3, 4, 6, 42, 49) and can be tracked with the specific antibody 17D1.

**Fig. 3.**
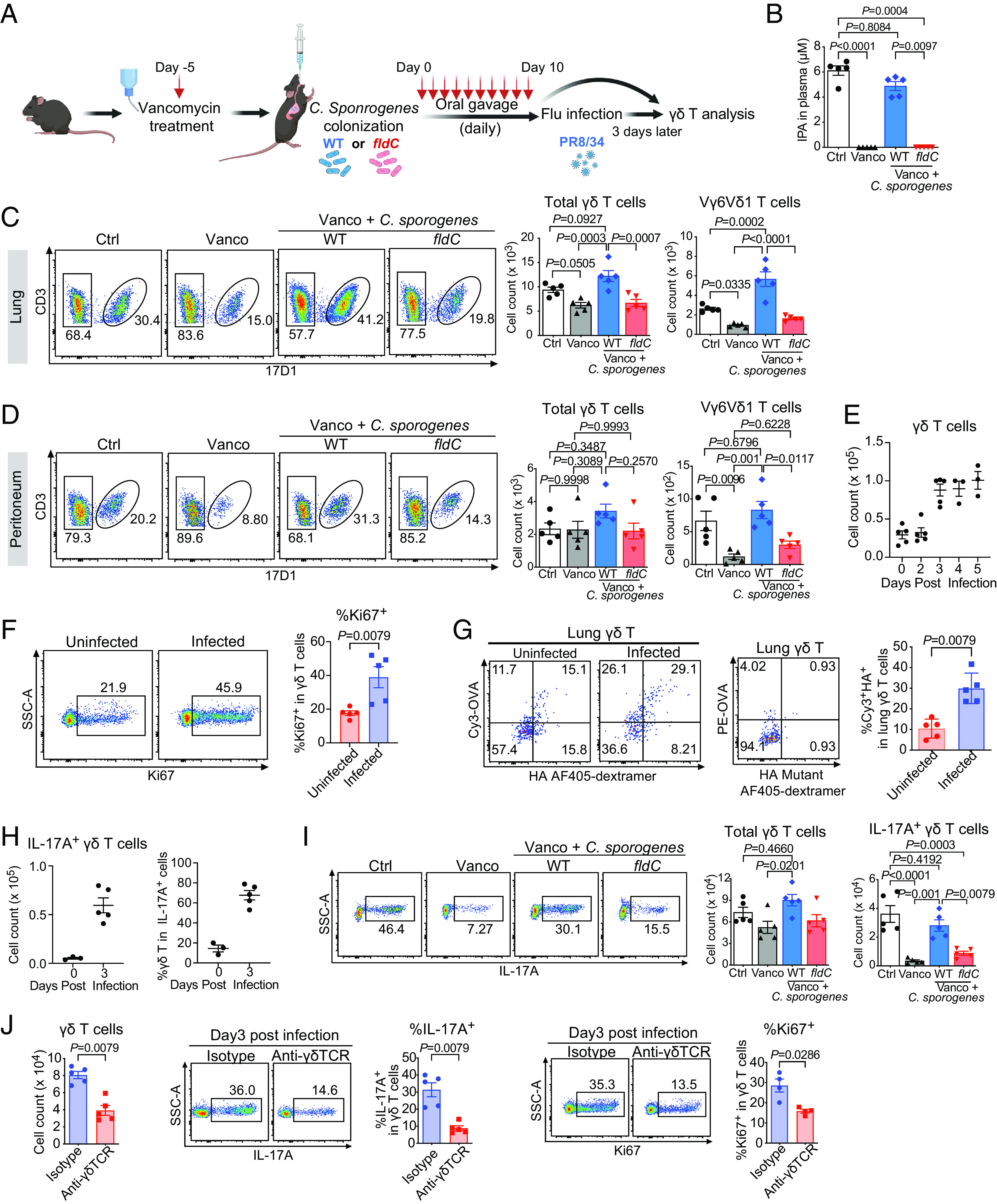
Commensal microbiota metabolite IPA maintains polyspecific innate-like Vγ6Vδ1 T cell homeostasis and response to infection. (*A*) Schematic of the experiment: C57BL/6 mice were treated with vancomycin, followed by WT or *fldC* mutant *C. sporogenes* colonization. Mice were either analyzed directly after bacteria colonization (*C* and *D*) or subjected to influenza virus infection and analyzed on day 3 after infection (*I*). (*B*) Plasma IPA level from mice of indicated groups as determined by liquid chromatography-mass spectrometry (LC–MS) measurement. Ctrl: mice without treatment, Vanco: mice treated with vancomycin, Vanco + *C. sporogenes*: mice treated with vancomycin followed by WT or *fldC* mutant *C. sporogenes* colonization. (*C* and *D*) A representative FACS plot of 17D1 (which identifies Vγ6Vδ 1 γδ T cells) staining of (*C*) lung and (*D*) peritoneal γδ T cells (*Left*), and the summary of total lung γδ T cell (*Middle*) and Vγ6Vδ1 (17D1^+^) γδ T cell (*Right*) numbers from vancomycin-treated mice after WT or *fldC* mutant *C. sporogenes* colonization. (*E*) Cell counts of γδ T cells in the lungs of C57BL/6 mice at indicated days after influenza virus infection. (*F*) A representative FACS plot of Ki67 staining (*Left*) and the summary of Ki67^+^ cell frequencies (*Right*) of lung γδ T cells from uninfected and influenza virus-infected mice at Day 3 after infection. (*G*) A representative FACS plot of Cy3 and HA staining of lung γδ T cells from uninfected and influenza virus-infected mice at Day 3 after infection (*Left*), PE-OVA and HA mutant staining of lung γδ T cells (*Middle*), and the frequencies of Cy3^+^HA^+^ cells among total γδ T cells in indicated groups (*Right*). Enriched lung γδ T cells were stained with Cy3-OVA (2 μg/mL), PE-OVA (2 μg/mL), HA coupled AF405-dextramer (1.35 μM), and HA mutant coupled AF405-dextramer (1.35 μM). (*H*) Cell counts of IL-17A-producing γδ T cells (*Left*) and the percentage of γδ T cells in total IL-17A^+^ cells (*Right*) in the lungs of C57BL/6 mice at indicated days after influenza virus infection. (*I*) A representative FACS plot of lung IL-17A^+^ γδ T cells (*Left*) and the summary of total γδ T and IL-17A^+^ γδ T cell numbers (*Right*) from mice treated as indicated in (*A*) followed by influenza virus infection for 3 d. (*J*) Cell counts of total lung γδ T cells (*Left*), and FACS analysis of IL-17A (*Middle*) and Ki67 (*Right*) staining of lung γδ T cells from isotype or anti-γδTCR (GL3) antibody treated TCRδ-GFP mice at day 3 after influenza virus infection. GFP^+^ cells were gated as γδ T cells. Results were shown as the mean ± SEM. Each data point represented the result from one mouse. The *P* values in *B*–*D* and *I* were determined using one-way ANOVA with Tukey’s multiple comparisons test. The *P* values in *F*, *G*, and *J* were determined using the Mann–Whitney test. The results were representative of at least 2 independent experiments.

IPA is mainly produced by Gram-positive bacteria such as *Clostridium sporogenes* (*C. sporogenes*), which are sensitive to the antibiotic vancomycin (34). Vancomycin-treated C57BL/6 mice (Fig. 3*A*) had no detectable level of plasma IPA (Fig. 3*B*) and showed a significantly reduced number of Vγ6Vδ1 cells in the lung and the peritoneum as determined with the specific antibody 17D1 staining (Fig. 3 *C* and *D*). Recolonization of these mice with wild-type (WT) *C. sporogenes*, but not with the *fldC* mutant restored plasma levels of IPA and the numbers of Vγ6Vδ1 γδ T cells (Fig. 3 *B*–*D*). *C. sporogenes fldC* mutant does not produce IPA because the *fldC* subunit of the phenyllactate dehydratase is genetically disrupted (34). In comparison to Vγ6Vδ1 γδ T cells, the number of total γδ T cells in the lung was affected much less, while total γδ T cells in the peritoneum showed no significant changes after vancomycin treatment (Fig. 3 *C* and *D*). These observations were consistent with the supposition that the IPA-mediated γδ T cell homeostasis is an antigen-driven event as ~10% of total lung γδ T cells were polyspecific (Fig. 3*G*) and ~3% of total γδ T cells in the peritoneum were IPA-specific (*SI Appendix*, Fig. S5*A*).

### Commensal Microbiota Metabolite IPA Promotes Polyspecific Vγ6Vδ1 T Cell Response to Influenza Virus Infection.

To test whether IPA regulates polyspecific γδ T cell response in pathological condition in vivo, we established an Influenza virus infection model where male C57B L/6 mice were infected with influenza H1N1 PR8/1934 (PR8). In response to the infection, lung γδ T cells increased in number, peaking at day 3 and showing signs of proliferation as evidenced by Ki67 expression (Fig. 3 *E* and *F*). Before infection, ~10% of lung γδ T cells were Cy3^+^HA^+^. At day 3 after the infection, ~30% of lung γδ T cells were Cy3^+^HA^+^ (Fig. 3*G*). This represented a ~ninefold increase of polyspecific γδ T cell numbers, as compared to the ~three fold increase in total γδ T cell numbers after the infection. At this time point, IL-17 producing γδ T cells increased and they made up ~70% of total IL-17A-producing cells in the lung (Fig. 3*H* and *SI Appendix*, Fig.S5*B*). scTCR determination showed that ~60% of the IL-17A producing cells at day 3 expressed Vγ6Vδ1 TCRs (Dataset S4).

We then initiated Influenza virus infection from C57BL/6 mice that were pretreated with vancomycin, followed by the recolonization with WT *C. sporogenes*, or the *fldC* mutant, and analyzed lung γδ T cells 3 d later (Fig. 3*A*). We found that recolonization of these mice with WT *C. sporogenes*, but not with the *fldC* mutant, restored the numbers of lung γδ T cells, and the IL-17A response after the infection (Fig. 3*I*). In this infection, TCR surface expression was required for γδ T cell response. Infection of *Tcrd*-green fluorescent protein (GFP) mice pretreated with an anti-γδ TCR antibody GL3 to induce γδ TCR internalization (54) resulted in much reduced proliferation and IL-17A response from γδ T cells (as identified by the GFP expression) (Fig. 3*J*). While WT mice survived the infection, mice treated with GL3 succumbed (*SI Appendix*, Fig. S5*C*).

These observations indicated that encountering antigen in the periphery is required for the polyspecific innate-like Vγ6Vδ1 Tγδ17 cells to function, underscoring the importance of recognizing multiple ligands in their function.

### Human Polyspecific γδ T Cells Increase in Frequency in Response to Immune Challenges.

**Fig. 4.**
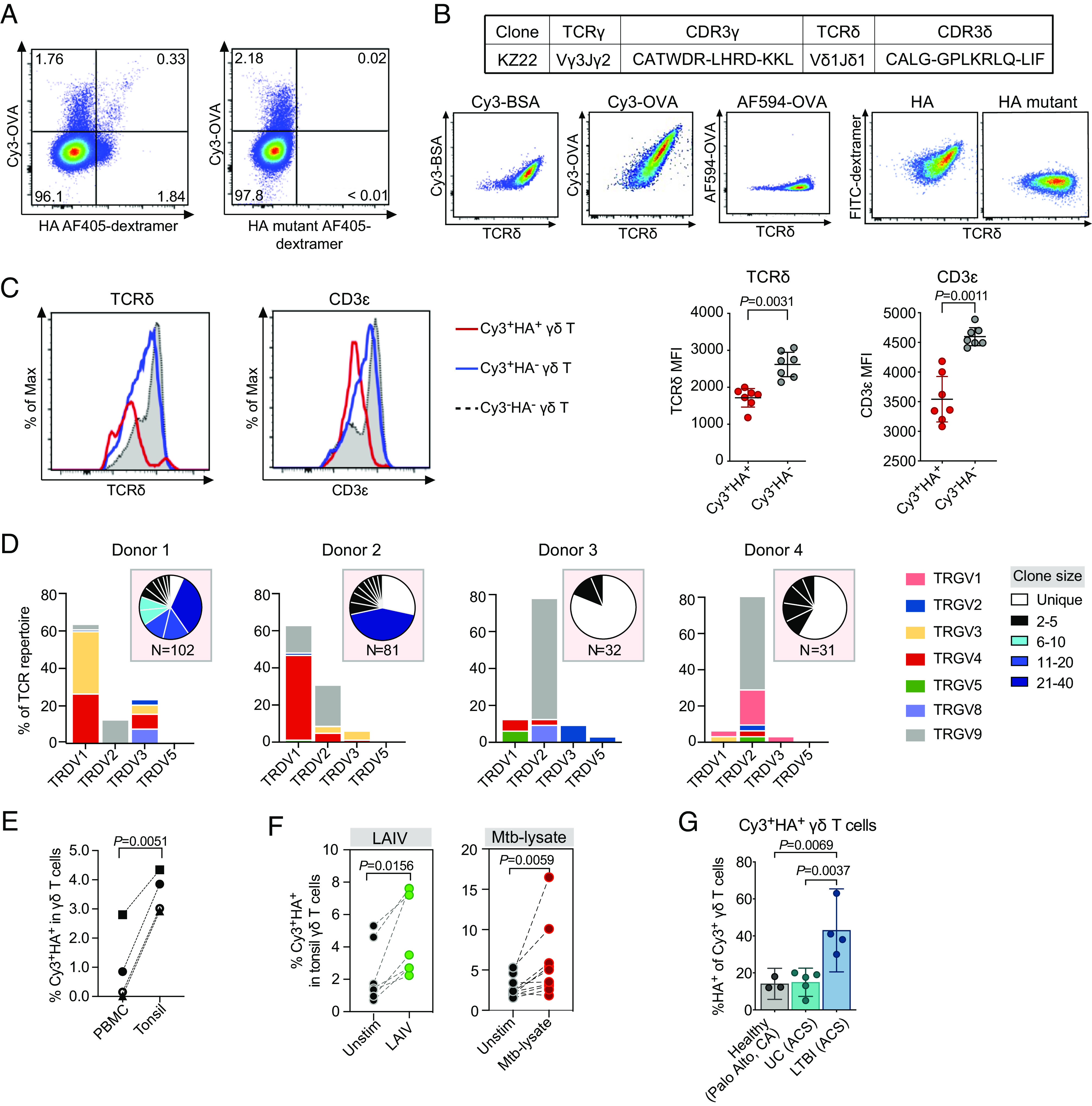
Human polyspecific γδ T cells respond to immune challenges. (*A*) A representative FACS plot of peripheral blood γδ T cells from a healthy donor stained with Cy3-OVA (60 μg/mL) and AF405 labeled SAv-dextramer coupled HA (*Left*) or HA mutant peptide (*Right*) (0.45 μM, SAv concentration). (*B*) The CDR3 sequences of KZ22 γδ TCR and FACS analysis of KZ22/Jurkat β^−^ cells stained with Cy3-BSA (5 μg/mL), Cy3-OVA (1 μg/mL), Alexa Fluor 594-OVA (1 μg/mL), and FITC labeled SAv-dextramer (0.45 μM, SAv concentration) coupled with HA or HA mutant peptides. The results were representative of at least three independent experiments. (*C*) Representative histograms comparing the expression levels of CD3ε and TCRδ on indicated γδ T cells. The results are representative of at least 3 independent experiments (*Left*). The mean fluorescent intensities (MFI) of CD3ε and TCRδ expression across different donors (*Right*). The *P* values were determined using the paired *t* test. Normality was assessed using the Shapiro–Wilk test. Error bars represented mean and 95% CI. (*D*) TCR V gene usage of Cy3^+^HA^+^ γδ T cells isolated from four blood bank PBMC samples and their corresponding clonal expansions, indicated in pie charts. (*E*) Frequency of Cy3^+^HA^+^ γδ T cells in total γδ T cells from PBMCs and tonsils of the same donor. The *P* values were determined using the paired *t* test. Normality was assessed using the Shapiro–Wilk test. (*F*) Frequency of Cy3^+^HA^+^ γδ T cells in tonsil organoids unstimulated or stimulated with LAIV (*Left*) or *Mtb*-lysate (*Right*). The *P* values were determined using the nonparametric Wilcoxon rank-sum test. (*G*) Frequency of Cy3^+^HA^+^ cells in local healthy donors and uninfected (UC) and *Mtb*-infected donors from a South African adolescent cohort (ACS). Cy3^+^ γδ T cells were gated to analyze the percentage of HA^+^ cells. Error bars represented mean and 95% CI. The *P* values were determined using one-way ANOVA with Tukey’s multiple comparisons test.

γδ TCRs show the most divergence when human and murine antigen receptor sequences are compared. Prevailing dogma has that human and murine γδ T cells have different antigen-specific repertoires. Nonetheless, one of the defining characteristics of adaptive immune recognition is that the antigen specificity, but not the antigen-specific receptor sequences, is conserved through evolution. The recognition of lysozyme by specific murine, human, and camel antibodies (55) as well as by the adaptive immune receptors of sea lamprey (56), and the recognition of PE by specific human, bovine, and murine γδ TCRs (23) are such examples. Indeed, human Cy3^+^HA^+^ γδ T cells were readily identifiable in peripheral blood mononuclear cells (PBMCs) by FACS staining, as exemplified by the identification of KZ22 TCR (Fig. 4 *A* and *B*).

In general, the frequencies of Cy3^+^HA^+^ γδ T cells among individuals were variable: ~1.5 to 30% of total γδ T cells in the PBMCs were Cy3^+^, and ~2 to 80% of the Cy3^+^ cells were also HA^+^ (*SI Appendix*, Fig. S6*A*), suggesting that the frequency of the polyspecific γδ T cells may reflect the immune experience of the individual. Nonetheless, compared to Cy3^+^HA^−^ or Cy3^−^HA^−^ γδ T cells, Cy3^+^HA^+^ γδ T cells exhibited lower CD3ε and TCRδ expression levels (Fig. 4*C*), which is indicative of prior antigenic exposure (57).

Directly ex vivo TCR determination of peripheral blood Cy3^+^HA^+^ γδ T cells from these donors showed that Cy3^+^HA^+^ γδ TCR repertoires were diverse and varied among individuals (Fig. 4*D*) (Dataset S5). Like the murine Cy3^+^HA^+^ γδ TCRs, some of the CDR3 sequences were enriched in positively charged amino acids, and others were enriched in residues that confer flexibility, such as Gly and Ser. They also contained Vγ9Vδ2 TCRs with the canonical CDR3γ sequence CALWEVQELGKKIKV, generated from the Vγ9 and JγP gene segments without N-nucleotide addition, or with limited VJ junctional variations. The same TCRγ sequences were described for fetal-blood-derived γδ T cells, which express cytokine receptors and have effector functions (58), and for γδ T cells with an “innate-like” transcription program (59).

Cy3^+^HA^+^ γδ T cells were present with variable frequencies in the tonsils (*SI Appendix*, Fig. S6*B*) but showed higher frequencies among tonsillar γδ T cells compared to paired peripheral blood γδ T cells (Fig. 4*E*). Stimulating tonsil organoid cultures with live-attenuated influenza vaccine (LAIV) induced Cy3^+^HA^+^ γδ T cell expansion when compared with unstimulated cultures. Cy3^+^HA^+^ γδ T cells also expanded after *M. tuberculosis* (*Mtb*) lysate stimulation (Fig. 4*F*). In addition, higher frequencies of Cy3^+^HA^+^ γδ T cells in total γδ T cells were present in the PBMCs of a South African adolescent cohort (ACS) with controlled *Mtb* infection than those from their healthy counterpart, or from blood center in Stanford, CA (Fig. 4*G*). Collectively, these observations indicated that human polyspecific γδ T cells also respond to different immune challenges.

## Discussion

Although the specificity of antigen recognition is central to an effective adaptive immune response, many individual B cell antigen receptors can recognize multiple ligands of different molecular properties, and individual αβ TCRs can recognize different MHC/peptide complexes (60). This degeneracy in antigen receptor recognition is complementary to focused antigen detection and is essential for host immune defense and is conserved among species. Here, through the investigation of the antigens recognized by a Cy3-specific γδ TCR, we find a population of γδ T cells that show polyspecificity. As this analysis is confined to γδ T cells with TCRs that bind Cy3 and HA, the overall polyreactivity of γδ T cells could be more extensive.

The fact that a relatively small number of lymphocytes can respond to a broad range of challenges is particularly relevant to γδ T cells’ function. It suggests that the antigen-specific repertoire of γδ T cells could be effectively much larger than previously estimated (~10^2^ to 10^4^) from the frequencies of individual antigen-specific γδ T cells which ranges between ~0.01% to a few (<2%) percent of the total γδ T cells (1). In fact, the γδ TCR polyreactivity that we observe here likely contributes to the high abundance of antigen-specific γδ T cells which would allow these cells to mount a response with magnitude similar to that of antigen-expanded αβ T cells (61, 62) despite the relative paucity of γδ T cell numbers. Antigen receptor polyspecificity/cross-reactivity also enables the development of activated/memory lymphocytes (63), such that a strong and rapid response can be elicited even when lymphocytes encounter an antigen for the first time. Indeed, providing rapid T cell responses in diverse settings is an important and unique γδ T cell function. We found that polyspecific γδ T cells were enriched for cells with an activated phenotype in naive animals and included the innate-like γδ T cells, the major regulators reported in various pathological/physiological conditions, including the responding γδ T cell population in the lung after influenza virus infection we described here. Furthermore, human γδ T cells with similar polyspecificity show enhanced response to immune challenges.

A hallmark of adaptive immune recognition is that antigen receptors with different sequences recognize the same target with different affinities, which allows them to mount a more flexible response. Different TCRs with the same polyspecificity may not recognize completely overlapping sets of antigens and are likely to have different affinities to their common antigens, and thus the ability to preferentially respond to different immune challenges. In this context, both the Vγ6Vδ1 TCRs and the Vγ1Vδ6.3 TCRs are Cy3^+^HA^+^, but Vγ6Vδ1 cells often appear at the onset of an inflammatory response, and Vγ1Vδ6.3 cells at the late stage of infection. It was reported that soluble Vγ6Vδ1 and Vγ1Vδ6.3 TCRs stained normal macrophages as well as many of the same mouse cell lines. However, after *Listeria* infection, the Vγ6Vδ1 TCR stained macrophages present early after infection, while macrophage staining by Vγ1Vδ6.3 TCRs increased over time (64).

γδ T cells differ from αβ T cells not only in antigen recognition requirement and antigen-specific repertoire, but also in the effector fate development and activation requirement (1). These features together with the γδTCR polyspecificity we discuss here suggest a way for γδ T cells to provide T cell response in a variety of settings in which αβ T cells could not respond to. Moreover, although γδ T cells recognize common B cell antigens and some antibodies are polyspecific, the effector function of antibody-mediated response and T cell–mediated response is different. A case in point is the recent work showing that CAR-T cells specific for CD19 are extremely effective clinically in treating lupus (65), whereas treatment with the same antibody was ineffective (66). Taken together, the unique characteristics of γδ T cells which allow important T cell functions to be induced by a much broader range of ligands and in a variety of physiological and pathological conditions suggest how γδ T cells contribute uniquely to host immune competence and conserved throughout vertebrate evolution.

## Materials and Methods

### Mice.

C57BL/6J mice and *Tcrd-*H2BEGFP mice (TCRδ-GFP) (stock no. 016941) were obtained from the Jackson Laboratories. Male mice (7 to 12 wk of age) were used for experiments. All mice were maintained under specific pathogen-free conditions at the Stanford Animal Facility. All experiments were performed following the Institutional Biosafety Committee’s guidelines, and all procedures and protocols were approved by the Institutional Animal Care and Use Committee. Throughout, mice were randomly assigned to the experimental groups.

### Human Samples.

Peripheral blood samples from healthy adult donors were obtained from the Stanford Blood Bank (Palo Alto, CA, USA). PBMC samples from a ACS who were either uninfected or with controlled *Mtb* infection were previously described (67). All eligible donors themselves gave written informed assent while their parents/legal guardians gave written, informed consent. The study protocols were approved by the Human Research Ethics Committee of the University of Cape Town. Individuals were classified as *Mtb*-infected based on a positive QuantiFERON TB Gold In-tube assay (Qiagen; >0.35 IU/mL). Tonsils samples were collected from children undergoing surgery for obstructive sleep apnea or hypertrophy at Stanford Hospital. Written informed consent was obtained from parents/legal guardians. Ethics approval was granted by the Stanford University Institutional Review Board.

### Tonsil Organoid Culture.

Tonsil organoids were established as previously described (68, 69). Briefly, whole tonsils (overall healthy, without obvious signs of inflammation) were collected in saline after surgery and then immersed in an antimicrobial bath of Ham’s F12 medium (Gibco) containing Normocin (InvivoGen), penicillin, and streptomycin for 1 h at 4 °C for decontamination. Tonsils were then briefly rinsed with PBS and manually disrupted into a suspension by processing through a 100 μm strainer with a syringe plunger and cryopreserved. Frozen cells were thawed, washed, enumerated, and then plated (6 × 10^6^ cells in 100 μL per well) into permeable (0.4 μm pore size) membranes placed in standard 12-well tissue-culture plates. Organoids were cultured for 7 d at 37 °C, 5% CO_2_ with or without *Mtb*-lysate (10 μg/mL, BEI Resources) or LAIV (1 μL per well, an equivalent of 1.6 × 10^4^ to 1.6 × 10^5^ fluorescent focus units per strain; FluMist Quadrivalent, Medimmune). Harvested cells were washed followed by flow cytometry analysis.

### Influenza Virus Infection Model.

Influenza A/PR/8/34 H1N1 was purchased from Charles River (Cat# 10100374, Lot# 4XP201023), aliquoted, and stored at −80 °C. Virus titer was determined with plaque assay. For each infection, a new vial of the virus was thawed. Male mice were anesthetized and intratracheally infected with diluted influenza virus in 20 μl PBS (1/10,000 dilution from 10^7^ pfu. per mL virus stock).

### Mouse *C. sporogenes* Recolonization.

WT and *fldC* mutant *C. sporogenes* ATCC 15579 were cultured at 37 °C in a Coy anaerobic chamber using a gas mix containing 5% hydrogen, 10% carbon dioxide, and 85% nitrogen, and the hydrogen level was maintained at 3.3% by anaerobic gas infuser. All media and plasticware were prereduced in the anaerobic chamber for at least 16 h before use. *C. sporogenes* strains were first streaked on Reinforced Clostridial Medium (RCM) plates. A well-isolated colony of each strain was inoculated in trypticase yeast extract medium or RCM broth (without agar). Bacterial overnight cultures (7 × 10^7^ CFU/mL) were mixed with glycerol (final 20%) and aliquoted into 2 mL prereduced cryovial tubes for further experiments. Conventional C57BL/6J mice were pretreated with vancomycin in the drinking water (0.5 g/L, Sigma) for 5 d, and then, WT or *fldC* mutant *C. sponrogenes* were transplanted by oral gavage for 10 consecutive days at 2.1 × 10^6^ CFU per mouse per day.

### Flow Cytometry.

For all flow cytometry experiments, cells were first incubated with Fc receptor blocking antibody binding (Mouse: 2.4G2 from BD, Human: TruStain FcX from BioLegend) for 20 min on ice and then stained with different antibody cocktails for another 30 min on ice. The following antibodies, coupled to the designated fluorochromes, were used in the study: anti-mouse TCRγδ (GL3), CD19 (6D5), CD11b (M1/70), TCRβ (H57-797), Gr-1 (RB6-8C5), CD3ε (145-2C11), CD121a/IL-1R (JAMA-147), IL-17A (TC11-18H10.1), CD11c (N418), F4/80 (BM8), CD27 (LG.3A10), CD44 (1M7), CD62L (MEL-14), CD45 (30-F11), TER119 (TER119), Ki67 (SolA15) antibodies; and anti-human CD19 (HIB19), TCRβ (IP26), CD14 (HCD14), TCRγδ (5A6.E9), CD3ε (UCHT1) antibodies, which are purchased from ThermoFisher Scientific, BD and BioLegend. For intracellular staining, cells were preincubated with protein transport inhibitors or cell stimulation cocktail (plus protein transport inhibitors) (ThermoFisher Scientific) for 3.5 h at 37 °C before surface staining. Cells were fixed and permeabilized with Cytofix/Cytoperm™ solution (BD), followed by staining with antibodies against intracellular antigens (4 °C for 30 min). For identifying Vγ6Vδ1 γδ T cells using 17D1antibody, cells were first stained with anti-TCRγδ antibody (GL3) and then incubated with supernatant from the 17D1 hybridoma followed by staining with anti-rat IgM (II/41, BD) as described (70). Flow cytometry was performed on the LSRII, LSRII.UV, and Symphony analyzer (BD), and data were analyzed using FlowJo software (Treestar). FACS sorting was performed on Falstaff or PICI machine (BD).

### Dextramer, Tetramer, and Other Antigen Conjugations.

One or more of the following reagents were used to stain γδ TCR transfectants, mouse or human primary γδ T cells, based on the experimental design.

#### Cy3 conjugates, 5-HIAA, and IPA conjugates.

Cy3-conjugated ovalbumin (OVA) (Sigma) and Cy3-conjugated chicken gamma globulin (Rockland) were prepared as previously described (29). 5-HIAA (Sigma) or IPA (Sigma) conjugated allophycocyanine (APC) (Prozyme), phycoerythrin (PE) (Prozyme), or BSA (Sigma) were generated using the Mannich condensation reaction. Briefly, 10 μM APC, PE, or BSA was mixed with 2.7 mM 5-HIAA or IPA in 0.25 M 2-(N-morpholino) ethanesulfonic acid (MES) buffer (pH 5.5) with 500 mM NaCl, 2 mM vitamin C (Sigma), and 3.5% final concentration of formaldehyde (plus 10% methanol to prevent polymerization) for 24 h at 37 °C. All procedures were protected from light. Unbound 5-HIAA or IPA was removed using a 10 KD cutoff spin column (Millipore). Control APC, PE, or BSA was made using the same conjugation protocol but without the addition of 5-HIAA or IPA.

#### Fluorescently labeled biotinylated peptide–streptavidin (SAv) dextramer.

Peptides were synthesized with a biotin tag attached to the aminohexanoic acid (Ahx) linker at the N terminus (ELIM BIOPHARM). The reaction was performed in PBS by mixing 1.75 μM peptide with 0.5 μM fluorescein isothiocyanate (FITC) (BD) or Alexa Fluor 405 (AF405) labeled SAv (ThermoFisher Scientific) and incubating for 30 min at room temperature. Then, 0.0083 μM biotin_60_-dextran (MW = 500 KD, Invitrogen) was added and incubated for another 30 min at room temperature. Unbound peptides were removed using a 10-KD cutoff spin column (Millipore).

#### Fluorescently labeled serotonylated peptide dextramer.

The serotonylation of small GTPase peptide Rac1 peptide (ELIM BIOPHARM) was carried out as described (71). Briefly, 50 mM biotinylated peptide was mixed with 0.3 μM human recombinant TG2 in a buffer (pH 7.4) containing 20 mM Tris, 25 mM HEPES, 150 mM NaCl with 10 mM CaCl_2_, and 1 mM serotonin (Sigma) and incubated overnight at 37 °C. The serotonylated peptide was purified by a Pierce™ C18 Spin Columns (ThermoFisher Scientific, 89870) and analyzed by electrospray ionization mass spectrometry (ESI-MS). Based on calculated and observed masses, the peptide was modified with a single serotonin moiety. The serotonylated peptide was then generated to a FITC-labeled dextramer as described above.

#### Fluorescently labeled Ym1 and hemagglutinin SAv tetramer.

Ym1 (Sino Biological) and hemagglutinin protein (cleaved from PR8 virus, a gift from Dr. Nicole Baumgart, (UC Davis) were first biotinylated with EZ-Link Sulfo-NHS-Biotin (ThermoFisher Scientific) and free biotin was washed off using a 10-KD cutoff spin column (Millipore). The protein concentration was determined by A280 using Nanodrop (ThermoFisher Scientific). Then, biotinylated Ym1 or HA protein was mixed with FITC-SAv (BD) or PE-SAv (ThermoFisher Scientific) at protein: SAv molar ratio of 4:1 and incubated 30 min at room temperature.

### FACS Analysis of Ligand Binding of γδ TCR Transfectants.

Murine γδ TCR expressing 58α^−^β^−^ cells or 58α^−^β^−^ NFAT reporter cells and human γδ TCR expressing Jurkat β^−^ cells were generated as described (23) (see *SI Appendix* *f*or details). To analyze antigen binding, cells were stained with antigen conjugates, LIVE/DEAD Aqua, and anti-TCRγδ antibody (GL3 for mouse γδ TCR, 5A6.E9 for human γδ TCR) for 1 h on ice. The staining concentrations are shown in figure legends.

### FACS Analysis of Primary γδ T Cell Antigen Binding.

#### Analysis of murine Cy3^+^HA^+^ γδ T cells.

Murine γδ T cells were negatively enriched as described (23, 42) (see *SI Appendix* *f*or details). Then, enriched cells were rested overnight in serum-free media (AIM V™ Medium, Gibco) and stained with Cy3-OVA (2 μg/mL) and Alexa Fluor 405-labeled HA peptide SAv-dextramer (1.35 μM) or PE-OVA (2 μg/mL) and Alexa Fluor 405-labeled HA mutant peptide SAv-dextramer (1.35 μM) at 37 °C for 1 h. Cells were also stained with APC/Cy7-conjugated anti-TCRβ, CD19, CD11b, CD11c, F4/80, and TER-119 antibodies, anti-TCRγδ, anti-CD3ε, anti-CD27, anti-CD44, anti-CD62L, and LIVE/DEAD Aqua at the final 20 min, following by flow cytometry or single cell sorting. Aqua- and APC/Cy7-positive cells were excluded from the analyses.

#### Analysis of human Cy3^+^HA^+^ γδ T cells.

Human γδ T cells were first negatively enriched from total PBMC or tonsils with APC-conjugated anti-TCRβ, CD19, CD14 antibodies and anti-APC MicroBeads (Miltenyi). The enriched cells were collected from unlabeled cells that passed through MACS column (Miltenyi) and then stained with Cy3-OVA (60 μg/mL), Alexa Fluor 405-labeled HA peptide SAv-dextramer (0.45 μM) on ice for 1 h. Cells were also stained with anti-TCRγδ, anti-CD3ε, and LIVE/DEAD Aqua (ThermoFisher Scientific). Aqua- and APC-positive cells were excluded from the analyses.

### TCR-Ligand Interaction in a Cell-Free System.

The soluble Vγ6Vδ1 TCRs were produced in BHK-21 cells and purified as described (23) (see *SI Appendix* *f*or details). The direct binding of soluble Vγ6Vδ1 TCR to multiple analytes was assessed by biolayer interferometry (BLI) using an Octet QK instrument (Pall ForteBio).

#### Soluble TCR-ligand interaction.

The analytes included OVA (Sigma), Cy3-OVA, BSA (Sigma), IPA-BSA, the recombinant Ym1 protein (Sino Biological), and recombinantly generated fusion-competent ectodomain HA protein. The binding of the recombinant influenza virus A/PR/8/34 hemagglutinin mobilized on the chip was confirmed with a hemagglutinin conformation-specific antibody (clone: C179) (72) to ascertain that the immobilized HA retained its conformation.

Soluble Vγ6Vδ1 TCR was captured onto the amine-reactive second-generation (AR2G) biosensors using the amine-reactive second-generation reagent kit. The ligand-bound biosensors were dipped into a decreasing concentration series (20 μM followed by twofold dilutions) of the indicated analytes in 1× PBST (1× PBS with 0.05% Tween-20) to determine the binding kinetics. Double referencing was performed using unliganded biosensors and an irrelevant αβ TCR (JM22). The soluble αβ TCR was produced in insect cells and purified as described (73) (see *SI Appendix* *f*or details). JM22 binds an influenza matrix protein-derived peptide (M1_58-66_-A*02:01). In addition, the signals from the Vγ6Vδ1 TCR bound biosensor in the OVA and BSA reference channels were used for nonspecific binding correction as appropriate. The traces were processed using ForteBio Data Analysis Software. The data were fitted globally to a simple 1:1 Langmuir interaction model.

#### Competition assay for evaluating small molecule binding to soluble Vγ6Vδ1 TCR.

Soluble Vγ6Vδ1 TCR was bound to AR2G biosensors using the amine-reactive second-generation reagent kit. The free sites on activated biosensor tips were blocked using 1 M ethanolamine (pH 8.5). The tips were washed with 1× PBST before incubating the ligand-bound biosensor tips with increasing concentrations of the indicated small molecules (1 to 75 mM) for 30 min. Then, the binding of Ym1 (15 μM) in the presence of the small molecule at the indicated concentration was measured. Vγ6Vδ1 TCR bound biosensor incubated only in 1× PBST (no small molecule treatment) was used to determine the maximal Ym1 binding. The fractional reduction in maximal Ym1 binding (*f*_max_) was calculated and plotted as a function of small molecule concentration. The data were fitted to a four-parameter logistic, sigmoidal function. Three independent experiments were performed.

### Induction of In Vivo Antibody-Mediated γδ TCR Internalization.

For antibody-induced TCR internalization, each mouse was administered 100 μg anti-TCR γδ antibody (GL3, produced and purified in the lab) or IgG isotype control (HTK888, BioLegend) with intraperitoneal injection. Experiments were carried out after 5 d.

### Statistical Analysis.

No samples or data points were excluded from the analysis. Statistical analyses were performed with GraphPad Prism Software version 9.3.1. Results represent the mean and error bars depict the SEMs or SD. The *P* values were determined as described in figure legends.

## Supplementary Material

Appendix 01 (PDF)

Dataset S01 (XLSX)

Dataset S02 (XLSX)

Dataset S03 (XLSX)

Dataset S04 (XLSX)

Dataset S05 (XLSX)

## Data Availability

Raw and processed scRNA-seq data that support the findings of this study have been deposited in the Gene Expression Omnibus under the accession number GSE251670 (74). Standard procedures for scRNA-seq data analysis were performed using Seurat 3.0 and the cutoff values for filtering, normalization, and variable gene selection are described in *SI Appendix*. All the other data are available in the main text and/or supporting information.
