# Supplementary material for: γδ T cell antigen receptor polyspecificity enables T cell responses to a broad range of immune challenges
Source: Proc Natl Acad Sci U S A. 2024 Jan 16;121(4):e2315592121. doi: 10.1073/pnas.2315592121 (PMC10823224; doi:10.1073/pnas.2315592121)
Supplement: Supplementary file 1 — Appendix 01 (PDF) [file pnas.2315592121.sapp.pdf]

Supporting Information for

γδ T cell antigen receptor poly-specificity enables T cell response to a broad range of immune challenges

Jing Guo[1, 2], Roshni Roy Chowdhury[1, 2]†, Vamsee Mallajosyula[3], Jianming Xie[1, 2]†, Megha Dubey[1, 2], Yuanyuan Liu[4], Jing Li[3], Yu-ling Wei[1, 2]†, , Brad A. Palanski[5]†, Conghua Wang[1, 2]†, Lingfeng Qiu[6, 7], Mané Ohanyan[1, 2]†, Oliver Kask[1, 2]†, Elsa Sola[3], Lilit Kamalyan[3], David B. Lewis[2, 8], Thomas Scriba[9], Mark M. Davis[1, 3, 10]*, Dylan Dodd[1, 4], , Xun Zeng[1, 6, 7, 11]*, Yueh-hsiu Chien[1, 2]*

Affiliations:

[1]Department of Microbiology and Immunology, Stanford University School of Medicine; Stanford, CA 94305, USA.

[2]Program in Immunology, Stanford University School of Medicine; Stanford, CA 94305, USA

[3]Institute for Immunity, Transplantation and Infection, Stanford University School of Medicine; Stanford, CA 94305, USA.

[4]Department of Pathology, Stanford University School of Medicine; Stanford, CA 94305, USA.

[5]Department of Chemistry, Stanford University; Stanford, CA 94305, USA.

[6]State Key Laboratory for Diagnosis and Treatment of Infectious Diseases, National Clinical Research Center for Infectious Diseases, The First Affiliated Hospital, College of Medicine, Zhejiang University; Hangzhou 310003, China.

[7]National Medical Center for Infectious Diseases, Collaborative Innovation Center for Diagnosis and Treatment of Infectious Diseases, The First Affiliated Hospital, College of Medicine, Zhejiang University; Hangzhou 310003, China.

[8]Department of Pediatrics, Stanford University School of Medicine; Stanford, CA 94305, USA.

[9]South African Tuberculosis Vaccine Initiative, Institute of Infectious Disease and Molecular Medicine and Division of Immunology, Department of Pathology, University of Cape Town, Cape Town, South Africa

[10]Howard Hughes Medical Institute, Stanford University School of Medicine; Stanford, CA 94305, USA.

[11]Research Units of Infectious disease and Microecology, Chinese Academy of Medical Sciences; Beijing, 100730, China.

*Correspondence: Mark M. Davis (mmdavis@stanford.edu), Xun Zeng (xunzeng@zju.edu.cn), and Yueh-hsiu Chien (chien@stanford.edu)

This PDF file includes:

Supporting text
Figures S1 to S6
Legends for Datasets S1 to S5
SI References

Other supporting materials for this manuscript include the following:

Datasets S1 to S5

Materials and Methods

Cell lines

All cell lines were cultured at 37°C, 5% CO_2 incubator unless otherwise stated. Phoenix-Eco cells used for retrovirus production, BHK-21 cells used for soluble γδTCR expression, and HEK-293T for lentivirus production are cultured in DMEM (Gibco) supplemented with 10% heat-inactivated Fetal Bovine Serum (FBS), 2 mM L-Glutamine (Gibco), 100 Units/mL Penicillin (Gibco), and 100 μg/mL Streptomycin (Gibco). FreeStyle 293-F cells for Ecto-HA protein are cultured in FreeStyle 293 expression media (Thermo Fisher Scientific). γδ TCR transfectants were cultured in RPMI1640 (Gibco) supplemented with 10% Fetal Bovine Serum (FBS), 2 mM L-Glutamine (Gibco), 100 Units/mL Penicillin (Gibco), 100 μg/mL Streptomycin (Gibco), 1mM Sodium Pyruvate (Gibco), 100 μM Non-Essential Amino Acids (Gibco), and 57 μM 2-Mercaptoethanol (Sigma).

The generation of γδ TCR transfectants

Murine TCR γ and δ chain gene fragments were cloned into pMSCV-P2 and Z4 retroviral expression vectors, respectively. Then the recombinant plasmids were transfected into the Phoenix-Eco cell line. The retrovirus in the supernatant was collected to infect $58\alpha^-\beta^-$ cells. Transfectants with the highest TCR expression were selected by FACS sorting. Human TCR γ and δ chain gene fragments were cloned into lentiviral constructs (nLV Dual Promoter EF-1a-MCS-PGK-Puro) and transfected into HEK-293T cells separately. The virus was collected after 72 hours and transduced into Jurkat β^- cells, which were selected for the highest TCR expression by FACS sorting. All γδ TCR transfectants were cultured in complete RPMI 1640 media.

The generation and purification of soluble TCRs

Soluble Vγ6Vδ1 γδTCRs. The extracellular domains of the γ and δ chains of Vγ6Vδ1 TCRs were cloned in frame with a gene encoding a Phinovirus protease

site, by acidic (TCRδ) or basic (TCRγ) leucine zipper and a (histidine)$_6$ tag in the pMSCV-P2 and Z4 retroviral expression vectors and expressed in BHK-21 cells. The soluble TCRs were purified from the supernatant with nickel-nitrilotriacetic acid (Ni-NTA) beads (Qiagen). The acidic and basic zippers were removed with PreScission Protease (GE Healthcare).

Soluble JM22 αβTCRs. The extracellular domains corresponding to the TCRα and TCRβ chains of JM22 TCRs were codon-optimized and cloned separately into a baculovirus expression vector to transfect Sf9 insect cells (Expression Systems). Then the soluble TCRs were purified from the supernatant with Ni-NTA beads (Qiagen).

FACS analysis of competitive Cy3-staining assay

NX7/58$\alpha^-\beta^-$ cells were first incubated with 10 mM indicated compounds for 30 min on ice and then stained with 1 μg/ml of Cy3-OVA and APC-conjugated anti-mouse TCRγδ antibody (GL3) for another 30 min on ice for FACS analysis.

In vitro stimulation assay of NX7 γδ TCR transfectants

NX7/58$\alpha^-\beta^-$ cells or NX7/58$\alpha^-\beta^-$ NFAT-GFP cells were stimulated overnight with plate-bound antigen-conjugates, the corresponding unmodified protein, or anti-CD3ε antibody (BD). Then the supernatant from NX7/58$\alpha^-\beta^-$ cells was collected for IL-2 detection using mouse IL-2 ELISA kit (ThermoFisher Scientific), while NX7/58$\alpha^-\beta^-$ NFAT-GFP cells were harvested for GFP expression by flow cytometry.

The enrichment of murine γδ T cells

Cells from different organs were stained with APC/Cy7-conjugated anti-mouse TCRβ, CD19, CD11b, CD11c, F4/80, and TER-119 antibodies and anti-APC MicroBeads (Miltenyi), and then applied onto MACS column (Miltenyi). γδ T cells were collected from passing through unlabeled cells.

Analysis of murine Cy3$^+$HIAA$^+$ and Cy3$^+$IPA$^+$ γδ T cells

Murine splenic γδ T cells were negatively enriched, rested, and stained as Cy3$^+$HA$^+$ γδ T cells described. For Cy3 and HIAA staining, γδ T cells were stained with Cy3-OVA (2 μg/ml) and HIAA-APC (4 μg/ml) or PE-OVA (2 μg/ml) and APC (4 μg/ml). For Cy3 and IPA staining, γδ T cells were stained with Cy3-OVA (2 μg/ml) and IPA-APC (30 μg/ml) or PE-OVA (2 μg/ml) and APC (30 μg/ml). The staining was performed at 37°C for 1 h, followed by flow cytometry.

Generation of fusion competent HA for assaying TCR-ligand interaction in cell-free system

Ecto-HA protein was generated by fusing the ectodomain of PR/34 HA at the C-terminus to a β-rich T4 fibritin trimerization motif 'foldon' (1), followed by a 15 amino acid biotinylation tag (AviTag) and a hexa-histidine affinity tag. The codon-optimized construct was cloned into a mammalian expression vector with a CMV/R promoter. The full-length Ecto-PR/34 HA sequence was obtained from the NCBI influenza database (GenBank Accession number: ABD77675.1). The construct was expressed in FreeStyle 293-F cell line (Thermo Fisher Scientific) as per manufacturer's recommendation. Briefly, the cells were maintained in FreeStyle 293 expression media (Thermo Fisher Scientific) and transfected with HA expression plasmid using FuGene (Promega). The protein was purified from the supernatant using Ni-NTA agarose resin (Qiagen), followed by size exclusion chromatography using a Superdex 200 column (GE HealthCare). The fusion-competent HA was generated by treating the purified Ecto-HA with trypsin (NEB, P8101S) at a 40:1 substrate:trypsin ratio and the solution was rotated at room temperature for 1-2 hr. The mixture was then run through the S200 column on the FPLC in PBS buffer. The appropriate peaks were collected and concentrated using the Amicon 10kD filter (Millipore), 50 μl aliquots were flash frozen and stored in -80°C.

LC-MS analysis of plasma IPA

Mouse plasma samples were diluted in LC-MS water in 1:1 ratio, and 10 μl diluted plasma was added to a 96-well V-bottom plate containing internal standard (phenylpropionic acid (D9); 50 μM, 20 μL). Ninety microliters of an acetonitrile/methanol mixture (3:1) were added to precipitate proteins and extract IPA, and the solution was mixed five times by pipetting. Then the plate was centrifuged at 5,000 *g* for 20 min at 4 °C. Thirty microliters of supernatant was transferred to a 96-well V-bottom plate and mixed with 15 μL 3-nitrophenylhydrazine (200 mM in 50% acetonitrile) and 15 μL *N*-(3-dimethylaminop'opyl)-*N'*-ethylcarbodiimide (120 mM in 6% pyridine). The plate was sealed with a thermoplastic elastomer sealing mat (Thermo Fisher Scientific cat. #AB-0566) and incubated at 40 °C, 600 rpm in a thermomixer for 60 min to derivatize IPA. The reaction mixture (10 μL) was then quenched with 190 μL 0.02% formic acid in 10% acetonitrile/water, the plate was sealed, and then subjected to LC-MS analysis.

LC-MS analysis was performed on an Agilent 1290 Infinity II UPLC equipped with an Agilent 6545XT Q-TOF equipped with a dual jet stream electrospray ionization source operating under extended dynamic range (EDR 1700 m/z) in negative ionization mode. Eluent A consisted of 0.1% formic acid (v/v) in water and eluent B consisted of 0.1% formic acid (v/v) in 100% methanol. Samples (10 μL) were injected via refrigerated autosampler into mobile phase and separation was achieved with Waters BEH C18 1.7-μm particle size C18 columns (2.1 × 100 mm) at 50 °C using dual binary pumps with alternating column regeneration. The analytical pump applied the following gradient at a flow rate of 0.3 mL min^{-1}: 0−11 min, 15% to 65% B, 11−11.1 min, 65% to 15% B, 11.1 min−12 min, 15% B. The regeneration pump applied the following gradient at a flow rate of 0.2 mL min^{-1}: 0−0.1 min, 15% to 99.9% B, 0.1−6 min, 99.9% B, 6−9 min, 99.9% to 15% B, 9−12 min, 15% B. Electrospray ionization parameters included a gas temperature of 300 °C, drying gas flow of 6 L min^{-1}, nebulizer pressure of 30 psi, sheath gas temperature of 300 °C and flow of 11 L min^{-1}, capillary voltage of 4000 V, and fragmentor voltage of 140 V. MS1 spectra were collected in centroid mode, and

peak assignments in samples were made based on comparisons of retention times and accurate masses from authentic IPA standard. A calibration curve was prepared for indole-3-propionic acid and its concentration in samples was quantified by isotope-dilution mass spectrometry using phenylpropionic acid (D9) as an internal standard.

Cell isolations from lung, liver, and small intestine

Euthanized mice were perfused via the right cardiac ventricle with PBS. Lungs, livers, and small intestines were then harvested.

Cell isolation from lung and liver. Single cell suspension of lung and liver was prepared using a gentleMACS™ octo dissociator (Miltenyi). Livers and lungs were dissected into gentleMACS™ C tubes (Miltenyi) containing 4 ml of a mixture of collagenase (25 μg/ml liberase™, Roche) and DNaseI (10 μg/ml, Sigma) in PBS + 2% FBS. Then organs were dissociated with pre-set program (lung: m_lung_02_01; liver: m_liver_01_02) using a gentleMACS™ Octo dissociator (Miltenyi) and incubated for 30 min at 37°C in a shaker (200 rpm). Digested organs were homogenized with the gentleMACS program (lung: m_lung_02_01; liver: m_liver_02_02) and 10 mM EDTA was added. Suspensions were passed through a 100 μm cell strainer. Red blood cells were lysed with Ack lysing buffer (ThermoFisher Scientific). Lung cells were resuspended in 4 ml 36% Percoll (GE Healthcare) and centrifuged at 2,000 rpm for 5 min at room temperature. Lung lymphocytes were harvested from the bottom and washed with PBS + 1%BSA for further experiments. To enrich lymphocytes, liver cells were resuspended in 40% Percoll (GE Healthcare), then loaded on the layer of 70% Percoll and centrifuged at 2,500 rpm for 30 min at room temperature. The interlayer cells were collected and washed with PBS + 1%BSA for further experiments.

Cell isolation from small intestine. Intestinal intraepithelial lymphocytes (IEL) and lamina propria lymphocytes (LPL) were isolated as reported (2). In brief, the fat and Peyer's patches were removed. The intestines were washed in PBS and cut into pieces. The intestines were shaken in PBS containing 1 mM DTT, 1 mM EDTA, and 1% BSA for 30 min at 37 °C. Then suspensions were passed through

a 70 μm cell strainer and collected to prepare IELs. The tissues were washed with complete RPMI1640 medium and digested in RPMI1640 containing Collagenase D (1 mg/ml, Roche), Dispase (0.5 U/ml, Corning), and DNase I (0.1 mg/ml, Sigma) at 37 °C for 30 min in a shaker (150 rpm). The cell suspensions from the enzyme digestion were harvested to prepare LPLs. To enrich lymphocytes, cell suspensions collected for IELs, and LPLs were then applied to a Percoll (GE Healthcare) gradient (40% Percoll on the top, 80% Percoll on the bottom) by centrifugation at 2,500 rpm for 25 min at room temperature. Lymphocytes were collected from the interphase and washed twice with PBS + 1% BSA for further experiments.

Direct ex vivo single-cell TCR determination

Single cell was sorted into 96-well PCR plates and TCR sequencing was performed as described (3, 4). Murine and human TCR sequencing primer sequences have been previously reported (4, 5). Murine splenic total γδ T cells, $Cy3^{+}HA^{+}$ γδ T cells, and $Cy3^{-}HA^{-}$ γδ T cells, and human $Cy3^{+}HA^{+}$ γδ T cells from PBMC were sorted as described above. To obtain single-cell TCR information of lung IL-17A-producing γδ T cells, IL-17A capture assay (Miltenyi) was performed on lung cells from influenza virus-infected C57BL/6 mice (day 3 after infection). Briefly, lung cells were resuspended in 80 μl RPMI1640 with 5% mouse serum per $1x10^{7}$ cells and incubated with 20 μl of IL-17A catch reagent for 5 min on ice. This was followed by adding warm media (37°C) with 5% mouse serum to dilute cells to $1x10^{6}$ cells/ml and incubated for 45 min at 37°C with slow rotation. After washing, cells were resuspended in 80 μl of cold PBS with 0.5% BSA and 2 mM EDTA per $1x10^{7}$ cells and incubated with 1% anti-CD16/32, 10% normal rat serum, 10% normal hamster serum for 5 min on ice. Then 20 μl of biotinylated mouse IL-17A detection antibody was added per $1x10^{7}$ cells and incubated for 15 min on ice. After washing, cells were stained with LIVE/DEAD Aqua, PE-SAv, PerCP/Cy5.5-conjugated anti-TCRγδ, PE/Cy7-conjugated anti-CD3ε, APC/Cy7-conjugated anti-TCRβ, CD19, CD11b, CD11c, F4/80, TER-119 antibodies. $Aqua^{-}$ and APC/Cy7-

positive cells were excluded from the analysis. Single IL-17A+ γδ T cells were sorted for TCR analysis.

Single cell RNA-seq gene expression quantification by Smart-seq2 and data analysis

Single cell RNA-seq of Cy3+HA+ γδT cells sorted from spleen of naïve mice was performed using the Smart-seq2 protocol with some modifications as reported (6, 7). Briefly, single cells were sorted into 96-well plates containing 5 μl lysis buffer (0.8 U/μl RNase Inhibitor (Clontech), ~5,000 molecules of ERCC (External RNA Controls Consortium) spike-in RNAs (Ambion), 0.08% BioUltra Triton X-100 (Sigma-Aldrich), 2 μM oligo-dT_{30}VN (Integrated DNA Technologies, 5′-AAGCAGTGGTATCAACGCAGAGTACT_{30}VN-3′), 2 mM Qiagen dNTP mix) in each well. Immediately after sorting, plates were sealed with aluminium seal (Axygen), centrifuged, flash frozen on dry ice, and then stored at -80 °C. Before reverse transcription, the plates were thawed on ice and lysed at 72 °C for 3 min. 5 μl reaction mix containing 10 mM DTT, 2 μM TSO (Exiqon, 5'-AAGCAGTGGTATCAACGCAGAGTGAATrGrGrG-3′), 20 U/μl SMARTScribe Reverse Transcriptase (Takara), 2 U/μl RNase Inhibitor (Clontech) and 2× First Strand Buffer was added to each well and reverse transcription was carried out by incubating wells on a thermal-cycler (Eppendorf) at 42 °C for 90 min, 10 cycles of 50 °C for 2 min, 42 °C for 2 min, and stopped by heating at 70 °C for 15 min. Subsequently, 15 μl of PCR mix containing 1.67× KAPA HiFi HotStart ReadyMix (Roche, KK2602) and 0.17 μM IS PCR primer (IDT, 5′-AAGCAGTGGTATCAACGCAGAGT-3′) was added to each well and second-strand synthesis was performed on a thermal-cycler (Eppendorf) by using the following program: 1) 98 °C for 3 min, 2) 22 cycles of 98 °C for 20 s, 67 °C for 15 s and 72 °C for 6 min, and 3) 72 °C for 5 min. 1 μl of the cDNA products were used for TCR PCR reaction. The remaining 24 μl cDNA products were subjected to purification by AMPure XP beads (Beckman Coulter) on the Biomek FX^P Automated Workstation (Beckman Coulter): 15.6 μl of Ampure XP beads (0.65×) were added to each sample and mixed by pipetting up and down thirty times; the

mixture were incubated at room temperature for 5 min to let the DNA bind to the beads; then the 96-well plate was placed on the magnet for 5 min, and the liquid was removed while samples were on the magnet; the beads were wash with 180 μl of 80% (vol/vol) ethanol solution twice and air dried on the magnet for 6 min; 25 μl of water was added to each well, mixed by pipetting up and down ten times, and incubated at room temperature for 3 min; the plate was placed on the magnet for 3 min and the supernatants were transferred to a new 96-well plate; finally, 2 μl of the supernatants were subjected to quality control using capillary electrophoresis on a Fragment Analyzer (Agilent Technologies) by Stanford Protein and Nucleic Acid Facility.

cDNA in 96-well plates was transferred into 384-well Low Volume Serial Dil. (LVSD) plates (TTP Labtech) and diluted to 0.16 ng/μl using a Mosquito X1 liquid handler (TTP Labtech). Illumina sequencing libraries were prepared as described previously (8) using a Mosquito HTS liquid handler (TTP Labtech). In brief, the tagmentation was carried out on 0.4 μl double-stranded cDNA using the Nextera XT DNA Library Preparation Kit (Illumina, FC-131-1096). Each well was mixed with 0.8 μl Nextera tagmentation DNA buffer (Illumina) and 0.4 μl Amplicon Tagment Mix (Illumina), then incubated at 55 °C for 10 min. The reaction was stopped by adding 0.4 μl Neutralize Tagment Buffer (Illumina) and centrifuging at room temperature at 3,000 g for 5 min. Indexing PCR reactions were performed by adding 0.8 μl of pre-mixed 5 μM i5 and i7 unique dual indexing primers (IDT, customized) and 1.2 μl of Nextera NPM mix (Illumina). PCR amplification was carried out on a C1000 Touch™ Thermal Cycler with 384-Well Reaction Module (Bio-rad) using the following program: 1) 72 °C for 3 min, 2) 95 °C for 30 s, 3) 12 cycles of 95 °C for 10 s, 55 °C for 30 s and 72 °C for 1 min, and 4) 72 °C for 5 min.

After library preparation, wells of each library plate were pooled using a Mosquito HTS liquid handler (TTP labtech). Pooling was followed by two purifications using 0.65× and 1× AMPure XP beads (Beckman Coulter), respectively. Library quality was assessed by Agilent 2100 Bioanalyzer and normalized to 5 nM. Libraries were sequenced on the Hiseq4000 Sequencing

System (Illumina) in Stanford Functional Genomics Facility, acquiring 150-bp paired-end reads.

Stanford Functional Genomics Facility extracted and generated FASTQ files for each cell, distinguished by the unique dual index adaptors. Reads were aligned to the mm10 genome using STAR v2.6.1d. Transcript abundance was quantified using HTSeq v0.5.4p5.

Standard procedures for filtering, log-normalization, variable gene selection, dimensionality reduction and clustering were performed using the Seurat 3.0 package (9). Briefly, cells with fewer than 800 detected genes, more than 5,000 detected genes or more than 15% mitochondrial genes were discarded. To make counts comparable among cells, gene counts were normalized to 10,000 reads per cell, then log-transformed. Following PCA dimensionality reduction, cells were clustered by running the Louvain algorithm and visualized using UMAP. Differential expression analysis was performed using the Wilcoxon rank-sum test implemented in the Seurat package's 'FindAllMarkers' function. Significantly differentially expressed genes were defined as those with log fold change > 0.5 and Bonferroni-corrected p-value < 0.05.

Fig. S1

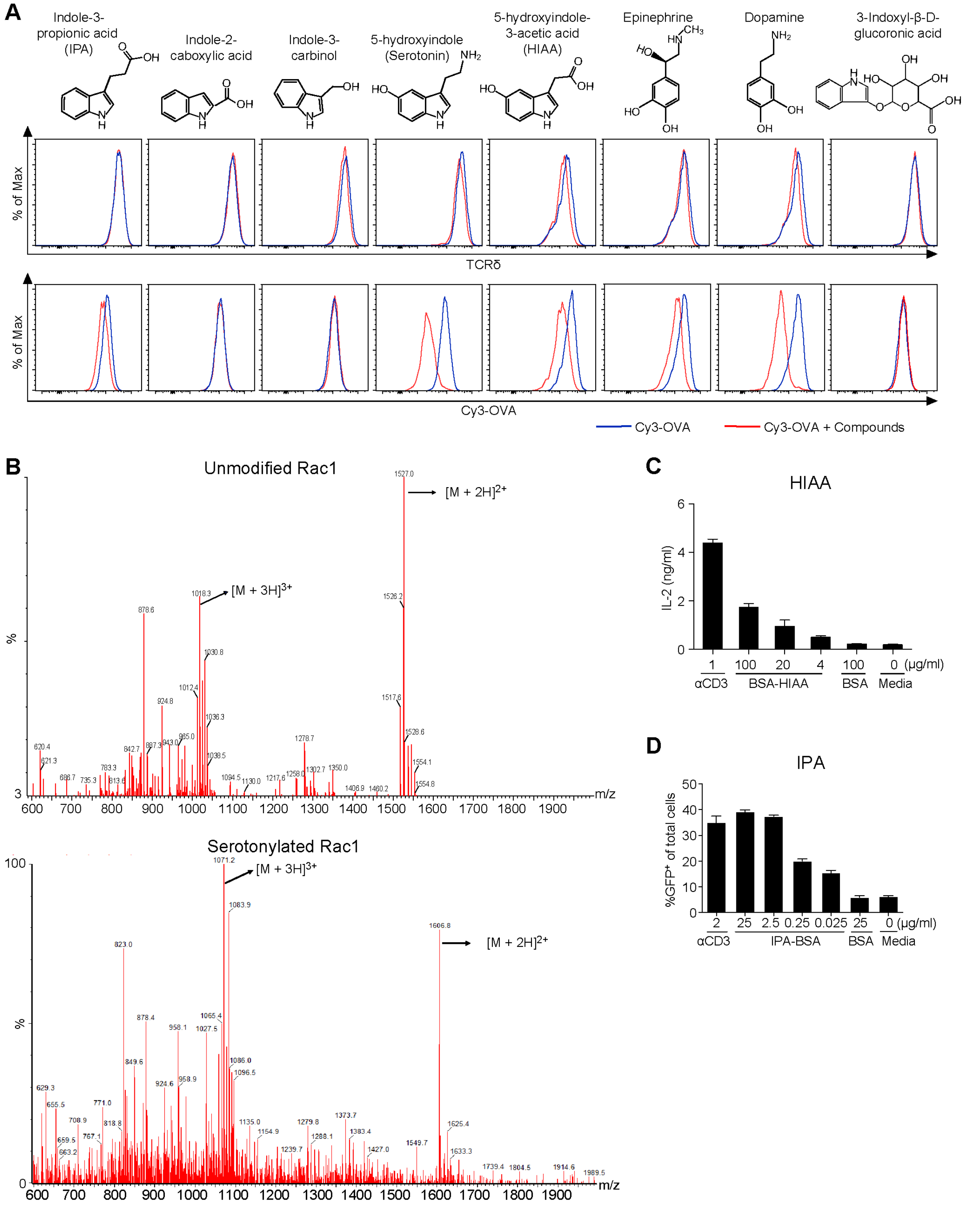

Fig. S1. NX7 γδ TCR recognizes indole containing bio-molecules related to Fig. 1.

(A) Histograms of TCRδ expression and Cy3-OVA staining intensity of NX7/58$\alpha^{-}\beta^{-}$ cells in the presence (red) and the absence (blue) of indicated small organic compounds. Data were representative of 3 independent experiments.

(B) Electrospray ionization mass spectrometry (ESI-MS) analysis of the unmodified (top) and the serotonylated (bottom) small GTPase peptides Rac1. The unmodified peptide: $M_{observed}$ = 3,052.0 Da ($M_{calculated}$ =3,051.0 Da); the serotonylated peptide: $M_{observed}$ = 3,211.6 Da ($M_{calculated}$ =3,209.2 Da). The mass spectrum of the unmodified peptide showed two major peaks at the mass-to-charge ratio (m/z) of 1527.0 and 1018.3, representing the doubly- and triply-charged GTPase peptide, respectively (top panel), and the serotonylated peptide showed two major peaks at the m/z of 1606.8 and 1071.2, representing the doubly- and triply-charged serotonylated peptide, respectively (bottom panel). Based on the observed molecular weight increase, the peptide was modified with a single serotonin moiety. The amino acid sequence of small GTPase peptide Rac1 was shown in Fig. 1b legend.

(C) IL-2 production from NX7/58$\alpha^{-}\beta^{-}$ cells stimulated with the indicated amounts of plate-bound anti-CD3ε, HIAA-BSA, and BSA.

(D) The percentage of GFP-positive cells in NX7/58$\alpha^{-}\beta^{-}$ NFAT-GFP cells after stimulation with the indicated amounts of plate-bound anti-CD3ε, IPA-BSA, and BSA.

The experiments in (C and D) were performed with each point of the assay in duplicates and the results were shown as the mean ± SEM from at least two independent experiments.

Fig. S2

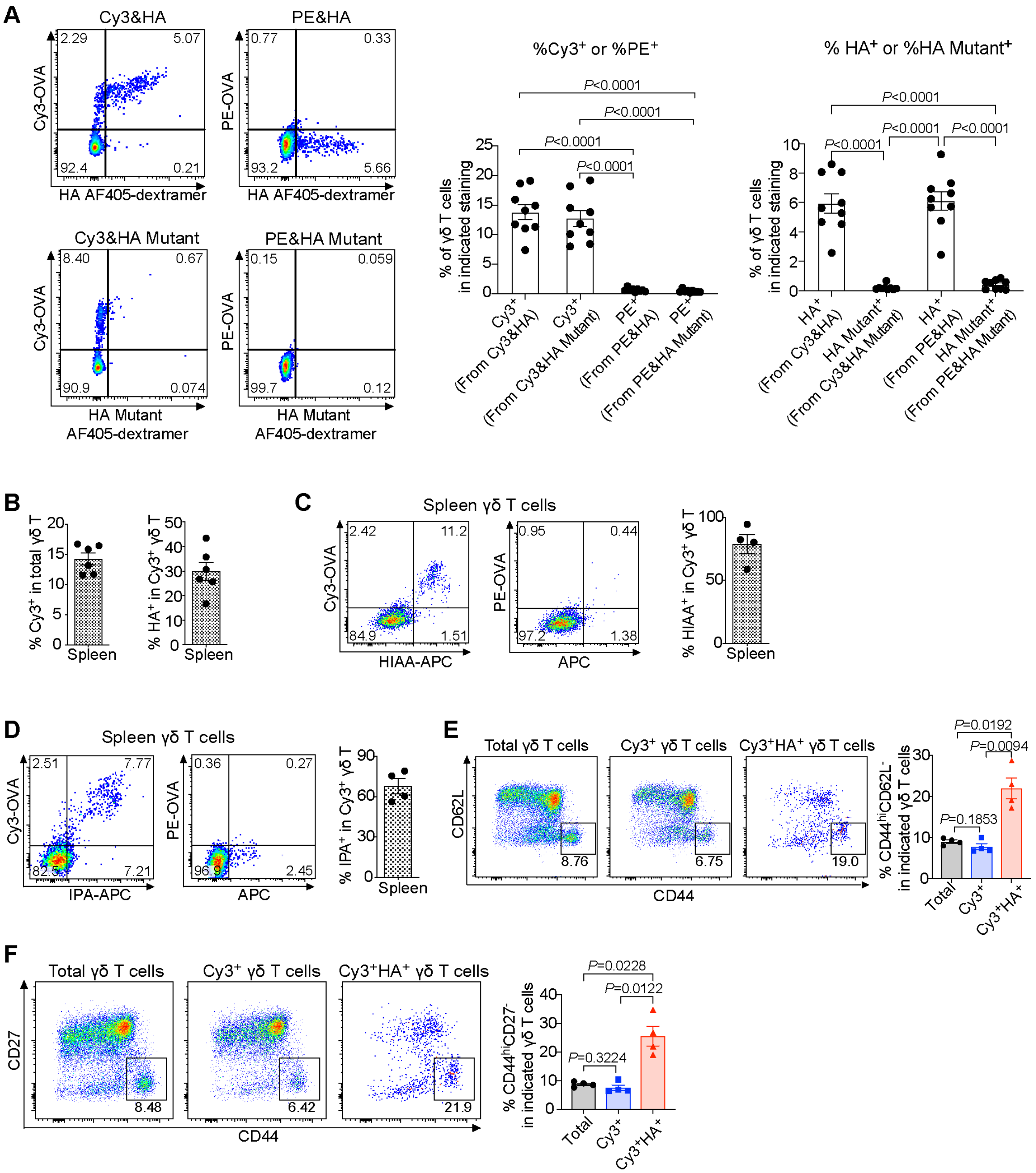

Fig. S2. Characterization of murine poly-specific γδ T cells, related to Fig. 2.

(A) A representative FACS plot of splenic γδ T cells from naïve C57BL/6 mice stained with indicated reagents (left) and the frequencies of $Cy3^+$ or PE^+ γδ T cells (middle) and HA^+ or HA mutant$^+$ γδ T cells (right) among total γδ T cells from indicated staining conditions.

(B) Frequencies of $Cy3^+$ γδ T cells among total γδ T cells (left) and HA^+ γδ T cells among $Cy3^+$ γδ T cells (right) from the spleens of naïve C57BL/6 mice.

(C, D) A representative FACS plot of splenic γδ T cells from naïve C57BL/6 mouse stained with (C) Cy3-OVA (2 μg/ml) and HIAA-APC (4 μg/ml) (left), PE-OVA (2 μg/ml) and APC control (4 μg/ml) (middle), and the frequencies of $HIAA^+$ γδ T cells among splenic $Cy3^+$ γδ T cells (right); (D) Cy3-OVA (2 μg/ml) and IPA-APC (30 μg/ml) (left), PE-OVA (2 μg/ml) and APC control (30 μg/ml) (middle), and the frequencies of IPA^+ γδ T cells among splenic $Cy3^+$ γδ T cells (right).

(E) A representative FACS plot of CD44 and CD62L staining on splenic total, $Cy3^+$, and $Cy3^+HA^+$ γδ T cells from naïve C57BL/6 mice (left) and the frequencies of $CD44^{hi}CD62L^-$ cells among indicated γδ T cell populations (right). Total, $Cy3^+$, or $Cy3^+HA^+$ γδ T cells were gated for the analysis.

(F) A representative FACS plot of CD44 and CD27 staining on splenic total, $Cy3^+$, and $Cy3^+HA^+$ γδ T cells from naïve C57BL/6 mice (left) and the frequencies of $CD44^{hi}CD27^-$ cells among indicated γδ T cell populations (right). Total, $Cy3^+$, or $Cy3^+HA^+$ γδ T cells were gated for the analysis.

Each data point represented the result from an individual mouse. Results were graphed as the mean ± SEM. The *P* values in (A, E, and F) were determined using one-way ANOVA with Tukey’s multiple comparisons test.

Fig. S3

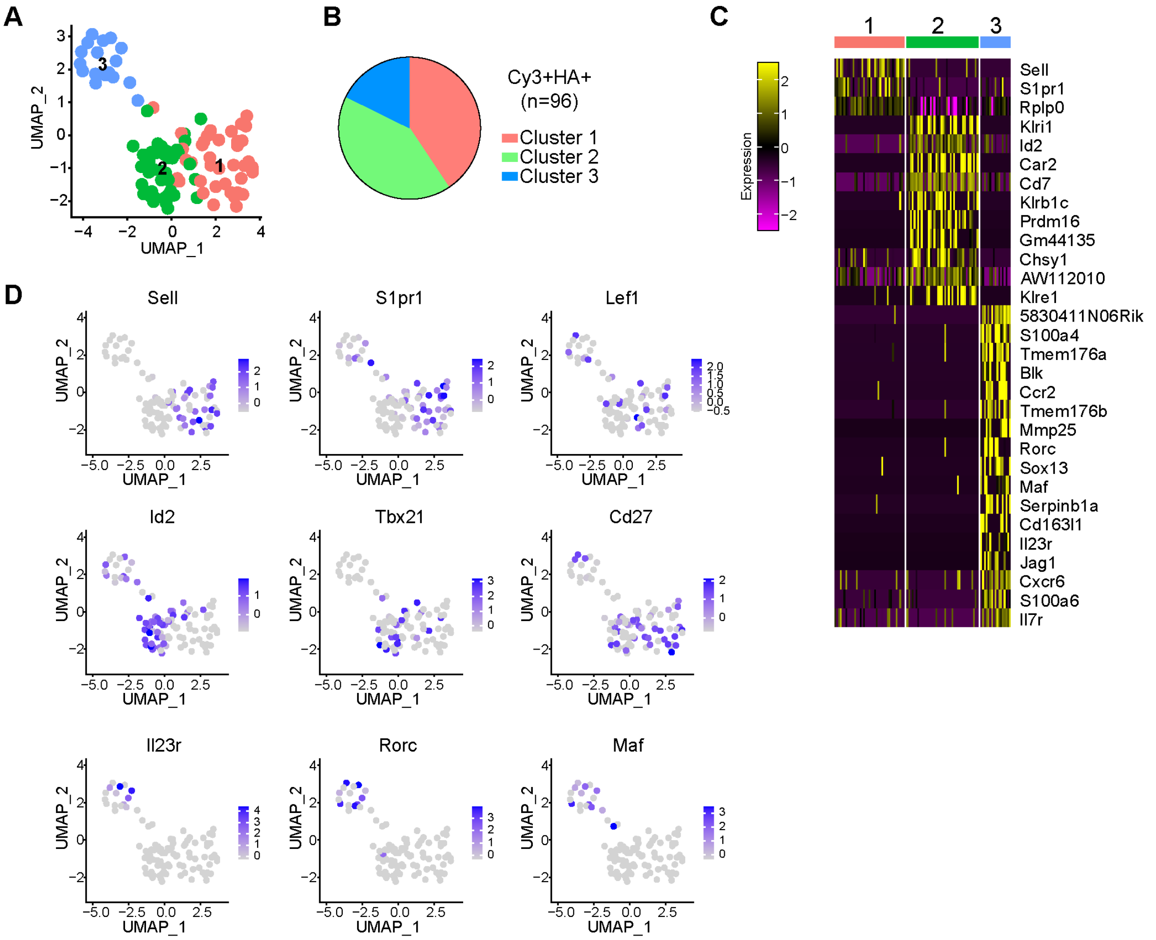

Fig. S3. scRNA-seq analysis of splenic Cy3$^+$HA$^+$ γδ T cells, related to Fig. 2. Splenic Cy3$^+$HA$^+$ γδ T cells from naïve mice were sorted for scRNA-seq using the Smart-seq2 protocol and analyzed using the R package “Seurat.”

(A) UMAP plot of the 3 subpopulations identified by unsupervised clustering based on the expression of marker genes in each cluster.

(B) Pie chart showing the cluster composition in Cy3$^+$HA$^+$ γδ T cells.

(C) Heatmap showing expression of genes differentially expressed in each cluster

(D) Feature plots showing gene expression of cluster-specific markers.

Fig. S4

Clone	TCRδ	CDR3δ	TCRγ	CDR3γ
BNT-9.12	Vδ6.3Dδ2Jδ1	CALWEL-WH-IGGIRA-TDKLVF	Vγ1Jγ4	CAVW- SGTSWVKIF

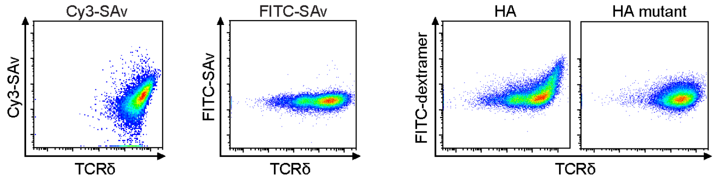

Fig. S4. The antigen binding of Vγ1Vδ6.3 γδ TCRs, related to Fig. 2.

The BNT-9.12 γδ TCR CDR3 sequences (top) and a representative FACS plot of BNT-9.12/58$\alpha^-\beta^-$ cells stained with Cy3-SAv (1μg/ml), FITC-SAv (5.2 μg/ml), HA or HA mutant peptide coupled with FITC SAv-dextramer (0.45 μM, SAv concentration). The results were representative of at least three independent experiments.

Fig. S5

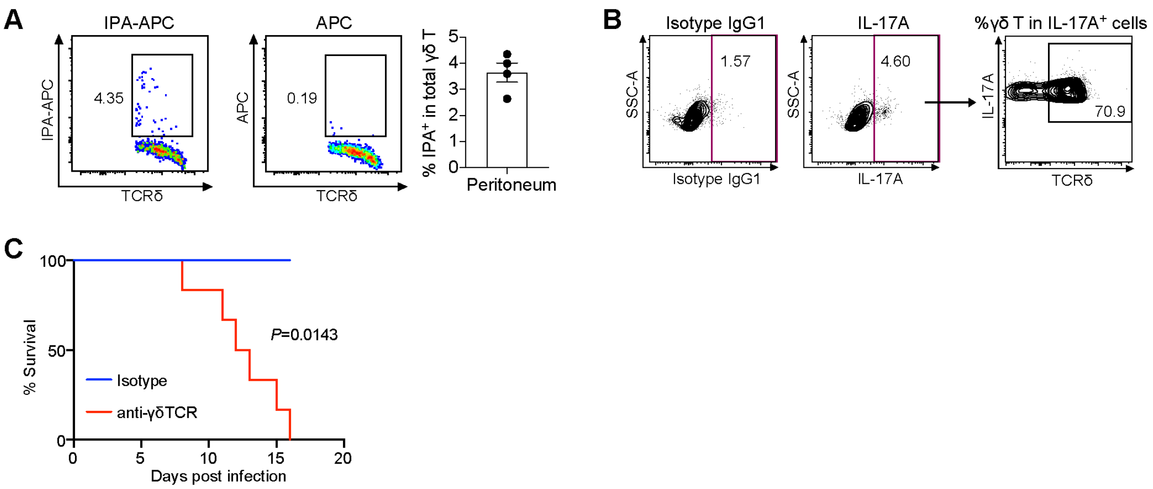

Fig. S5. IPA staining of peritoneal γδ T cells and lung γδ T cell response after influenza virus infection, related to Fig. 3.

(A) A representative FACS plot of peritoneal γδ T cells from naïve C57BL/6 mouse stained with IPA-APC (10 μg/ml) (left) or APC control (10 μg/ml) (middle), and the frequencies of IPA$^+$ γδ T cells among total γδ T cells (right). Each data point represented the result from an individual mouse. Results were graphed as the mean ± SEM.

(B) Gating strategy of γδ T cells in lung IL-17A$^+$ cells as shown in Fig. 3h. Aqua$^-$ CD45$^+$ cells were first gated for the analysis of IL-17A$^+$ cells, followed by the analysis of γδ T cells within the IL-17A$^+$ population.

(C) Survival curve of influenza virus infection of anti-γδTCR antibody (GL3) or isotype control antibody treated C57BL/6 mice (n = 6 per group). The *P* values were determined using the log-rank (Mantel-Cox) test.

The results were representative of at least 3 independent experiments.

Fig. S6

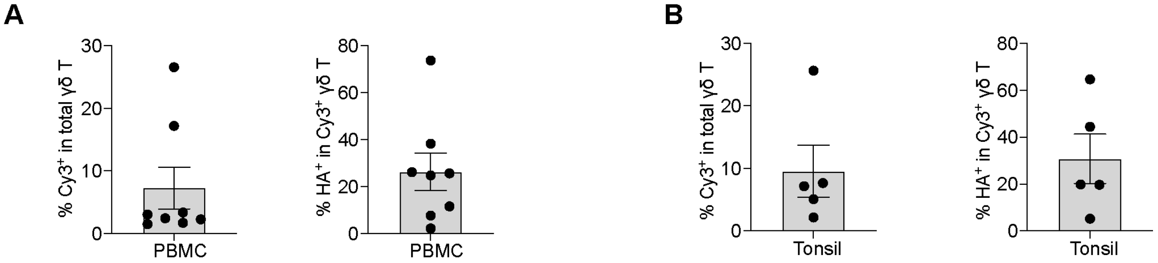

Fig. S6. The frequencies of indicated γδ T cell populations in human PBMCs (A) and tonsils (B), related to Fig. 4.

Dataset S1 (separate file). TCR sequences of splenic $Cy3^{+}HA^{+}$ γδ T cells from naïve C57BL/6 mice.

Dataset S2 (separate file). TCR sequences of splenic $Cy3^{-}HA^{-}$ γδ T cells from naïve C57BL/6 mice.

Dataset S3 (separate file). TCR sequences of total splenic γδ T cells from naïve C57BL/6 mice.

Dataset S4 (separate file). TCR sequences of murine IL-$17A^{+}$ γδ T cells from influenza virus-infected lung (Day3 after infection).

Dataset S5 (separate file). TCR sequences of human $Cy3^{+}HA^{+}$ γδ T cells from peripheral blood samples of healthy donors.

SI References

1. V. V. Mallajosyula *et al.*, Influenza hemagglutinin stem-fragment immunogen elicits broadly neutralizing antibodies and confers heterologous protection. *Proc Natl Acad Sci U S A* **111**, E2514-2523 (2014).
2. S. Sun *et al.*, Bifidobacterium alters the gut microbiota and modulates the functional metabolism of T regulatory cells in the context of immune checkpoint blockade. *Proc Natl Acad Sci U S A* **117**, 27509-27515 (2020).
3. A. Han, J. Glanville, L. Hansmann, M. M. Davis, Linking T-cell receptor sequence to functional phenotype at the single-cell level. *Nat Biotechnol* **32**, 684-692 (2014).
4. Y. L. Wei *et al.*, A Highly Focused Antigen Receptor Repertoire Characterizes gammadelta T Cells That are Poised to Make IL-17 Rapidly in Naive Animals. *Front Immunol* **6**, 118 (2015).
5. R. Roy Chowdhury *et al.*, NK-like CD8(+) gammadelta T cells are expanded in persistent Mycobacterium tuberculosis infection. *Sci Immunol* **8**, eade3525 (2023).
6. S. Picelli *et al.*, Full-length RNA-seq from single cells using Smart-seq2. *Nat Protoc* **9**, 171-181 (2014).
7. J. Li *et al.*, KIR(+)CD8(+) T cells suppress pathogenic T cells and are active in autoimmune diseases and COVID-19. *Science* **376**, eabi9591 (2022).
8. S. Darmanis *et al.*, A survey of human brain transcriptome diversity at the single cell level. *P Natl Acad Sci USA* **112**, 7285-7290 (2015).
9. A. Butler, P. Hoffman, P. Smibert, E. Papalexi, R. Satija, Integrating single-cell transcriptomic data across different conditions, technologies, and species. *Nat Biotechnol* **36**, 411-420 (2018).
